# SMS education for the promotion of diabetes self-management in low & middle income countries: a pilot randomized controlled trial in Egypt

**DOI:** 10.1186/s12889-017-4973-5

**Published:** 2017-12-19

**Authors:** Haitham Abaza, Michael Marschollek

**Affiliations:** Peter L. Reichertz Institute for Medical Informatics, Hannover Medical School, Carl-Neuberg-Str. 1, 30625 Hannover, Germany

**Keywords:** mHealth, Diabetes, SMS, Mobile phones, Self-management, Education, Glycemic control, HbA1c, Awareness, Text messaging

## Abstract

**Background:**

Due to the ubiquity of mobile phones in low and middle income countries, we aimed to examine the feasibility of SMS education among diabetic patients in Egypt, and assess the impact of educational text messages, compared to traditional paper-based methods, on glycemic control and self-management behaviors.

**Methods:**

We conducted a 12-week randomized controlled trial at Misr University for Science & Technology hospital in Cairo-Egypt. Known as MUST diabetes awareness program, patients were included if they had diabetes, owned a mobile phone, and could read SMS messages or lived with someone that could read for them. Intervention patients received daily messages and weekly reminders addressing various diabetes care categories. We expected greater improvement in their glycemic control compared to controls who only received paper-based educational material. The primary outcome was the change in HbA1c, measured by the difference between endpoint and baseline values and by the number of patients who experienced at least 1% reduction from baseline to endpoint. Key secondary outcomes included blood glucose levels, body weight, treatment and medication adherence, self-efficacy, and diabetes knowledge. Data were analyzed using ANCOVA, chi-square, and t-tests.

**Results:**

Thirty four intervention and 39 control patients completed the study. Over 12 weeks, 3880 messages were sent. Each intervention patient received 84 educational and 12 reminder messages plus one welcome message. Our primary outcome did not differ significantly (Δ 0.290; 95% CI -0.402 to 0.983; *p* = 0.406) between groups after 3 months, demonstrating a mean drop of −0.69% and −1.05% in the control and intervention group respectively. However, 16 intervention patients achieved the targeted 1% drop versus only 6 controls, suggesting clear association between study group and 1% HbA1c reductions (chi-square = 8.655; df = 1; *p* = 0.003). Secondary outcomes seemed in favor of intervention patients at endpoint, with considerable improvements in treatment and medication adherence, self-efficacy, and knowledge scores. Participants also indicated full satisfaction with the program.

**Conclusions:**

SMS education is a feasible and acceptable method for improving glycemic control and self-management behaviors among Egyptian diabetics. However, whether it is more effective than traditional paper-based methods needs further investigation.

**Trial registration:**

ClinicalTrials.gov NCT02868320. Registered 9 August 2016. Retrospectively registered.

**Electronic supplementary material:**

The online version of this article (10.1186/s12889-017-4973-5) contains supplementary material, which is available to authorized users.

## Background

Diabetes is a chronic condition associated with high levels of sugar in the blood [1]. According to the World Health Organization (WHO), the number of people with diabetes has nearly quadrupled, rising from 108 million in 1980 to 422 million in 2014 [2, 3], with Type 2 accounting for 90% of all cases [4]. Its prevalence and burden of disease have been rising worldwide, more rapidly in low and middle income countries (LMICs), mainly due to obesity and lack of physical activity [2, 3]. It is currently the eighth leading cause of death in the world and it is expected to become the seventh by 2030 [2, 5]. In 2012, it was the direct cause of 1.5 million deaths, more than 80% of which occurring in LMICs [2, 6]. It is also one of the four main non-communicable diseases (NCDs) and largest contributors to mortality in the WHO’s Eastern Mediterranean Region (EMR), causing more than 1.7 million deaths every year together with cardiovascular diseases, cancers, and chronic respiratory diseases [7].

Six of the top 10 countries in the world with highest diabetes prevalence are in the EMR [8]. Fact sheets and figures of the region show that the number of people with diabetes will nearly triple between the years 2000 and 2030. Of 22 countries, Egypt comes in 2nd place in terms of the number of diabetes cases [9], with national statistics showing that 17% of Egyptian adults are diabetic [10]. According to the International Diabetes Federation (IDF), the country had over 7.8 million adults with diabetes in 2015, a number that already exceeds the 2030 expectations [11]. Though there is good evidence that diabetes and its complications can be prevented or delayed by following a healthy diet, regular physical activity, adhering to medications and screening tests, maintaining a normal body weight, and avoiding tobacco [2, 3], people are rarely aware of the impact such behaviors could have on their risk of developing complications [10]. Consequently, over 60% of Egyptian diabetics receive no treatment due to lack of awareness or lack of availability of regular checkup [10].

Proper diabetes education can lead patients themselves to better manage their disease and successfully avoid complications. However, health systems cannot control all the factors that influence a person’s overall health‚ as doctors are not able to constantly monitor what their patients eat or whether they take their medications on time [12]. In Egypt, although patient education is part of the Ministry of Health (MOH) hospital accreditation scheme, it is not widely implemented (personal communication with Prof. Mahi Al Tehewy^1^). It traditionally takes place in the outpatient clinics via brief discussions on complications, medications, follow-up, healthy diet‚ and physical activity. However, factors such as transportation availability, distance, time, examination costs, or health awareness may affect the regular attendance of outpatient appointments [13]. Moreover, patients often indicate difficulty abiding to healthy lifestyles due to irregular working hours, food cravings, or lack of motivation to exercise. They are also prone to forget their doctor’s advice or possibly ignore it after leaving the clinic (personal communication with Dr. Amira El Ansary^2^). Therefore, a method that can easily reach them wherever they are, educate or give them regular tips about their disease, and provide the knowledge and motivation necessary for proper disease management could be beneficial. Further, knowledgeable patients might save doctors critical amounts of time in the clinic and spare the need for lengthy or redundant discussions.

Mobile technology presents an easy and effective way to reach a larger population since mobile phones have exceptionally exceeded other communication infrastructures in LMICs. According to the Ministry of Communications and Information Technology (MCIT), up to January 2016 there were 94.16 million mobile subscriptions in Egypt as opposed to 6.35 million fixed line subscriptions, with penetrations reaching 107.41% and 7.35% respectively. Further, there were 19.78 million mobile internet users vs. 3.8 million ADSL subscriptions in the same month. It is important to note that only 21.01% of mobile subscribers were mobile internet users [14], which in the authors’ opinion, could be a reflection of the proportion of smartphone owners in the country. Therefore, in contrast to mobile internet or smartphone apps, SMS messages can provide a simple way of communication and have the advantage of reaching a higher percentage of the population since they are supported by all types of mobile phones.

To our knowledge, prior to the time of preparation for the study, SMS technology might have been used in some EMR countries such as Saudi Arabia to provide health appointment reminders, yet not for health education. In Egypt, SMS messages had not been widely used in the healthcare field. Diabetes education might have been offered by some organizations via lectures, workshops‚ or pamphlets, but not through mobile text messages. They were also not used by public or teaching hospitals to communicate with their patients. Very few private hospitals had the possibility of sending their patients a text message, yet only in cases of appointment cancellations. The idea of SMS messages with educational content was regarded as promising, especially that it could aid hospitals meet the standard of patient education, one that is required by quality systems in Egypt (personal communication with Prof. Mahi Al Tehewy^1^ and Dr. Hani Farouk^3^).

On the national level, a non-governmental organization called Sukar Mazboot (Arabic for Diabetes Controlled) formerly announced they were in preparation of the first diabetes telephone hotline service in Egypt [15]. Further, the WHO’s regional office in Cairo announced in February 2016 the mDiabetes program, in collaboration with the International Telecommunication Union (ITU), and Egypt’s MOH, MCIT, and Ministry of Higher Education (MOHE). As part of a global initiative to reduce the burden of NCDs known as “Be He@lthy Be Mobile”, the program aims to empower diabetics to manage their condition and increase access to information on diabetes management. Targeting 700,000 patients, the first phase will start with 54 messages and 10,000 patients whose mobile numbers are already saved in national health insurance databases. The message content was reviewed by global WHO experts and comprises lifestyle choices and tips on living with diabetes and avoiding its complications [10, 16, 17].

This paper presents the design, implementation‚ and findings of our 3-month randomized controlled trial (RCT) that took place at the teaching hospital of Misr University for Science & Technology (MUST) in Cairo Egypt. Referred to as MUST diabetes awareness program (DiabAwPro), the study aimed to examine the feasibility of SMS education among Egyptian diabetic patients, and assess the impact of unidirectional educational text messages on their glycemic control and ability to self-manage their diabetes. Intervention group patients received daily SMS messages and reminders, and were expected to have greater improvement in glycemic control as opposed to patients of the control group who only received paper-based educational material. All participants were invited to attend interviews, complete questionnaires, and undergo follow-up tests throughout the study.

## Methods

### Study design

This was a 12-week randomized controlled intervention study. Patients were randomized and divided into two groups:

- An intervention group that received diabetes educational SMS messages in addition to reminder prompts to take tests and record readings.
- A control group that received no SMS messages.
- Both groups, however, received a booklet of diabetes care instructions at the beginning of the study. The booklet was meant to introduce intervention patients to diabetes management before receiving short SMS messages on the subject. It also intended to make control patients feel that they belonged to the program and encourage them to stay through the end of the study.
- Both groups also received a monitoring table to record their blood glucose measurements and return it after completion of the study.

Educational SMS messages were sent on a daily basis. Intervention patients received one message per day; each day from a different category. This allowed for a variety of information to be sent and covered seven message categories (diet, physical activity, complications, etc.) throughout the week. After 12 weeks, each patient had received 12 messages from each category making a total of 84 educational messages per patient.

Patients were monitored by the hospital’s outpatient clinic of internal and general medicine. They were invited to measure their blood glucose once a week according to a preset schedule, and take the HbA1c test at the beginning and end of the study period. As an incentive, all tests and measurements were provided free of charge. Further, patients were permitted to see the clinic’s doctor when necessary without paying any admission fees. A free dose of diabetes medications was planned to be offered to those who complete the study, should extra incentives come to need. Follow-up interviews and feedback questionnaires were also conducted throughout and after the study period. Blinding was only applicable to the outcome assessors (lab and clinic nurses), but to participating patients, the study remained unblinded.

### Study location and team

The Souad Kafafi Memorial Medical Center is the teaching hospital of Misr University for Science & Technology, shortly referred to as MUST hospital. It is located on the university campus in 6th October city, about 30 km from the center of Cairo. The hospital’s public section provides teaching and research opportunities in addition to low cost medical services. Our study took place in the outpatient clinic of internal and general medicine, which operates every day except Fridays and admits up to 40 patients per day. Besides the study’s researcher (primary author), the study team comprised a diabetes specialist, internal medicine doctors, and clinic and lab nurses.

### Recruitment

Upon receiving approval from both the MUST hospital Director and the head of internal medicine in April 2014, the researcher was authorized to attend patient examinations in the clinic and identify appropriate candidates for the study. The clinic worked daily from 9 am to noon and accepted patients on a first come first serve basis through a small admission system and a low-priced examination ticket. Patients visited the clinic for multiple and various reasons, one of which was diabetes and its complications. Upon entry of a diabetic patient, the researcher had a short interview with them, mainly checking compliance with inclusion criteria and obtaining personal data, number of diabetes years‚ and medications prescribed. The researcher also explained the study briefly and obtained verbal consent from patients to call and invite them to come back later for signing official documents and receiving formal introduction to the study. However, this method of recruitment did not prove success as it was very slow and did not attract many patients. Over 20 days, a total of 206 patients were seen, only 27 of which were diabetic. Moreover, on some days, none of the patients seen had diabetes.

In June 2014, the ethical review board (Protocol # 2014/3) was contacted and approvals were obtained from the hospital and university management to announce and promote our diabetes program via paper ads and flyers. An enrollment/consent form was also prepared and kept in the general medicine clinic. Ads were spread throughout the hospital and university buildings, thus allowing recruitment of visiting patients as well as diabetic hospital and university staff members, and providing a bigger variety of participants to eliminate potential bias. The ad read: “FOR DIABETIC PATIENTS: MUST hospital announces the start of its free diabetes awareness and monitoring program. If interested, please fill out the application form at the general medicine clinic”. Nurses at the clinic were instructed to obtain a completed and signed form from applicants complying with the study criteria. Patients were included if they had diabetes, owned a mobile phone, and were able to read SMS messages or lived with someone that could read for them. Patients were excluded if they could not read or were not SMS familiar and lived alone.

Data collected via enrollment forms included name, age, sex, address, occupation, mobile number, social status, diabetes years, reading ability, ability to open and read SMS messages, and whether there was someone at home that could read the messages if needed. Patients’ signatures and consent to participate were also obtained. Further, patients were informed that they would be contacted near the beginning of the study to complete a questionnaire, attend an interview‚ and take a baseline test. Recruitment went on with this method until October 2014, targeting a sample size of 80 in addition to 20 extra patients to account for dropouts if any. In November 2014, we started checking the enrollment forms for data validation and calling patients to complete and clarify any missing or misleading information (e.g. same phone number on multiple forms, both yes and no boxes checked, etc.). Consequently, recruitment was extended through the end of 2014 to replace patients who could not be reached due to incorrect contact information on their forms. Replacement also proceeded till March 2015 simultaneously with baseline interviews and tests to substitute for patients who failed to attend or were excluded during the interview.

### Baseline HbA1c testing, interview, and pre-study questionnaire

Between January and March 2015, patients were contacted to complete the pre-study questionnaire (see Additional file 1: Pre-study questionnaire) as part of a 30-min baseline interview, during which the baseline weight and blood glucose level were recorded and their recent test results (if any) were checked. Patients were also informed that they would be contacted again within a month to take a baseline HbA1c test and receive an instruction booklet on diabetes. As mentioned earlier, new patients were still being recruited to replace those who did not attend the interview, or those who were revealed by the interview to not comply with our selection criteria. Based on time availability, some of these patients completed both the enrollment form and the pre-study questionnaire, while others completed the enrollment form and were invited to come back for the interview a few days later. However, to avoid any further loss of patients, we emphasized on when and how they would be contacted, requested them to save the program’s phone number in order to recognize it when we call, and asked them to call us back if they couldn’t answer at the time of the call. We also encouraged them about the next steps of the study and stressed on their importance in monitoring their diabetes.

As of March 1st 2015, patients were invited to come back to the clinic for baseline HbA1c testing. They were contacted in the same order they attended the baseline interviews, i.e., those who had the interview first were contacted for baseline testing first. We invited 10 to 15 patients to come per day and gave appointments between 10 am and 1 pm every 15 min. Upon arrival of a patient at the clinic, the lab nurse collected the HbA1c sample then the patient joined the researcher to get a brief description of the next stage. During this short interview, all patients received a diabetes instruction booklet and a monitoring table in which they were instructed to fill their blood glucose readings and weight every week over 12 weeks. They were also asked to choose a week day (from Saturday to Tuesday) for taking the measurements‚ and were given the option to take them on that fixed day at the hospital free of charge, at home if they owned a glucometer, or at a nearby pharmacy and record the result. Further, they were advised that visiting the internal medicine doctor within the next 3 months would be free of charge for program participants, and were given the program’s mobile number to call in case of inquiries or if they wished to know the results of the baseline test without having to come to the hospital. Baseline testing was scheduled to last for 2 weeks.

As patient replacement was still ongoing, new participants were requested to complete the enrollment form and the pre-study questionnaire, then the HbA1c test was done and the booklet and monitoring table were provided. Replacements continued until a patient list was submitted for randomization on March 5th. After this date, patients that failed to attend their HbA1c testing appointment were not replaced, and new patients who asked to enroll were politely informed that the study had completed its required sample. As baseline testing was scheduled to end on March 14th, all patients were informed that the SMS messages would start within 2 weeks and that they might or might not get them according to randomization. Patients were also advised to read and follow the instruction booklet and commit to the SMS recommendations in case they received any. A full list of baseline HbA1c test results was obtained from the hospital lab on March 18th 2015.

### Interventions

The SMS message sending started on March 21st 2015 for 12 consecutive weeks. Patients were greeted first with a welcome message that read “Welcome to MUST diabetes awareness program! Please follow the instructions in order to keep your blood glucose levels normal”. Seven SMS categories, comprised of 12 messages each, were prepared with the objective of sending one category message per week day. The categories included educational, interventional‚ and lifestyle messages and were extracted from a publication of the WHO EMR office on diabetes education [18], in combination with the standards of the Egyptian MOH on patient education (personal communication with Prof. Mahi Al Tehewy^1^) as follows:

1. Diabetes knowledge and effects on social and personal life
2. Healthy diet
3. Physical activity
4. Smoking, foot care and diabetes complications
5. Medications and side effects
6. Tests and blood glucose measurement
7. Hyper- and hypoglycemia

Reminder messages were also sent to remind patients to take their blood glucose and weight measurements at the preset times. In order to encourage patients to expect and wait for the messages every day, category messages were sent daily at 11 am, while reminder messages were sent 4 days a week to corresponding patients at 11:15 am, 1 day ahead of the fixed measurement day. The message read “Do not forget to check your blood glucose level and weight tomorrow and record the result in your monitoring table”. This was to allow patients who wished to take the measurement at the hospital 24 h to make arrangements for their visit. The messages were sent using an online paid SMS service known as “Bulk SMS”, which featured message scheduling and bulk SMS sending through uploading a text file containing the receivers’ mobile numbers. Therefore, a text file with all intervention patients was used to send category messages daily, while 4 other files were prepared for the subset of patients to be reminded on Fridays, Saturdays, Sundays, and Mondays. The sender was set to “DiabAwPro” and the acronym was printed as a logo on the instruction booklet so that patients would recognize it upon receiving the messages. The researcher’s mobile number was also added to the text file to ensure the SMS messages were being sent and delivered daily. SMS sending proceeded until June 12th 2015.

The content of the instruction booklet (see Additional file 2: Instruction booklet) and the SMS messages (see Additional file 3) was developed to include information on our seven categories. However, due to the extra level of detail contained in the booklet, some categories were split to avoid compressing too much information under one category, and preserve readability and attractiveness. Information was mainly extracted from the websites of the American Diabetes Association (ADA) [19, 20], the Egyptian Society for Diabetic Care (Sukar Mazboot) [21], and two WHO EMRO publications [18, 22]. These choices were selected due to the popularity of the ADA guidelines among doctors at MUST, and the well written diabetes educational information on the Sukar Mazboot website, which had been translated to the Arabic language from the website of the French Diabetes Association A.J.D. For the purpose of our intervention, extracted information was shortened, assigned to the different categories, and presented in the form of easy and quick to read bullets in the instruction booklet. Further, for a friendly and attractive design, pictures representative of every category as well as colors were introduced.

As the booklet was ready, the 12 most important and non-redundant bullets were selected from each of the seven categories. They were further shortened and rearranged in a logical sending order to form straightforward text messages that fit the SMS character limit and easily grasp attention when read on a mobile phone’s screen. A nutrition leaflet (see Additional file 2: The proposed diet leaflet) was also prepared and attached to the booklet. The leaflet was developed specifically for our program from a combination of nutrition pamphlets that were already being used by the clinic and handed out to patients. The leaflet contained a proposed nutrition regimen for the three main daily meals‚ with exact portions, items to eat without reservation, and prohibited items specified. All content was translated, reviewed‚ and approved by our local diabetes specialist.

### Final HbA1c testing, interview, and post-study questionnaire

As Ramadan was starting on June 18th 2015, we decided to begin the final interviews and HbA1c tests 2 weeks ahead of schedule. Ramadan is a month during which people fast from dawn till sunset. Despite fasting, the month is known in Egypt for its variety of traditional foods and sweets whose abundance could easily lead to increased blood glucose levels, weight gain, and other diabetes risks. Therefore, people with diabetes should optimally visit their doctors before the beginning of Ramadan to adjust medication doses and times according to the special lifestyle of the month. People usually eat two main meals per day; one at sunset and one before dawn. Snacks and sweets are also available between meals and before bedtime. At the clinic, the daily pace becomes slower and most patients avoid making appointments while fasting especially in the hot summer weather. In light of the aforementioned lifestyle changes, final tests and interviews began on May 30th 2015 after consultation with our diabetes specialist, and ran until June 23rd 2015.

The post-study questionnaire (see Additional file 1: Post-study questionnaire) was prepared and printed. Patients were contacted by phone and invited to come in the same order they attended their baseline testing to ensure a period of 3 months between both tests. However, in order to allow intervention patients to receive the most number of messages prior to their final test, control patients were invited to come first. Following the same procedure of baseline testing, 10–15 patients were invited to come per day. Upon patient arrival, the final HbA1c test was done and the blood glucose level and weight were measured. The patient then met with the researcher for the final interview and completion of the post-study questionnaire, which mainly checked improvement in reported problems at baseline in addition to the patient’s opinion of the program. During this 30-min interview, the monitoring table was also viewed and collected by the researcher, and patients were advised to continue monitoring their blood glucose levels for their own sake the same way they were trained in the program.

As SMS sending ended, a final message was sent from “DiabAwPro” to all intervention patients that had not attended the interview. The message read “The SMS intervention is finished. Please come to the clinic for the final interview and HbA1c test. Wishing you a happy Ramadan! Tel: 01001665753”. Remaining control patients were also sent a similar message from the program’s mobile number asking them to call or come to the clinic for the final test. This was to ensure that all patients who were not answering their phones or not showing up for appointments were approached and reminded by all possible means of communication. We had attempted to reach them by SMS and phone calls for 3 weeks before we considered them dropouts. Final HbA1c lab results were received on July 29th 2015.

### Outcome measures

The primary clinical outcome of the study was the change in HbA1c levels, measured by the difference between endpoint and baseline values and by the number of patients who experienced a reduction of at least 1% from baseline to endpoint. Random blood glucose levels and body weight were also investigated as secondary clinical measures. Non-clinical secondary outcomes included treatment and medication adherence, diabetes self-efficacy, diabetes knowledge, rate of hospital/ER visits and stays, average frequency of blood glucose measurement, rate of regular exercise, patients’ confidence in healthcare provider, patient satisfaction, and healthcare provider’s reputation.

Our pre- and post-study questionnaires were created from a variety of diabetes education, knowledge, and assessment surveys [23–30], and translated into Arabic under the supervision of our diabetes specialist. Assessment methods were embedded in both questionnaires, and their items were used to produce scores to compare outcome measures between groups before and after applying the intervention. Treatment adherence was assessed using the Diabetes Self-Care Inventory (SCI) [31], which contains 14 items that measure patient perceptions of their adherence to diabetes treatment recommendations on a 5-point scale. The questions address four different domains, namely blood glucose regulation, insulin and food regulation, exercise, and emergency precautions. For the purpose of the study and as recommended by our diabetes specialist, the question concerning wearing a medic alert was removed as it is not widely used in Egypt. Further, the ketone testing item was replaced by a more comprehensive question addressing urine, lipids, and kidney function tests as well as eye examination. Question scores were averaged to produce an overall treatment adherence score for each patient.

Medication adherence was assessed by the Morisky Medication Adherence Scale (MMAS-4) [32, 33], a 4-item generic scale that assesses patients’ medication-taking behavior. The items are in the form of yes/no questions, with yes indicating a score of 0 and no indicating a score of 1. The scores of the four questions were summed to produce the patient’s overall medication adherence score. The Stanford Self-Efficacy for Diabetes [34] combined with the Michigan Diabetes Empowerment Scale-Short Form (DES-SF) [35] were used to measure diabetes self-efficacy (DSE). The form comprises 8 items that assess patients’ confidence in their ability to manage their diabetes on a 5-point scale. Similar to SCI, the scores of all questions were averaged to calculate the patient’s overall DSE score. Diabetes knowledge was assessed by 8 questions prepared by the researcher and the diabetes specialist based on what our patients needed to know and in reference with the Michigan Diabetes Knowledge (DKN) Scale [36, 37]. The items were scored on 3 levels‚ with 0, 0.5, and 1 indicating no knowledge, some knowledge, and full knowledge respectively. Question scores were averaged to produce an overall knowledge score for every patient in the range of 0 to 1.

The rest of the outcomes were measured by direct questions on both the pre- and post-study questionnaires. For instance, the rate of hospital/ER visits and stays was measured by asking patients how many times they were admitted to a hospital or an emergency room because of their diabetes in the last 3 months. Moreover, the average frequency of blood glucose measurement was assessed by one of the SCI questions, asking patients how often they usually checked their blood glucose at baseline, and instructing them to check at least once a week during the intervention period. Patient responses were scored on a 5-point scale and the number of patients that performed weekly measurements was recorded for each study group.

The rate of regular exercise was measured by another SCI item addressing how often patients engaged in physical activity, and an additional question checking whether they walked at least 30 min a day. Walking was chosen as it is a form of exercise that can be practiced anywhere, anytime, and without incurring any costs on patients. Patients’ confidence in their healthcare provider was measured by one 5-level question, while satisfaction was assessed by two questions on a 5-point scale. The scores of both questions were averaged to produce an overall patient satisfaction score with their healthcare provider. Further, a patient’s opinion section was developed by the researcher and added to the post-study questionnaire to assess patients’ satisfaction with the program, and whether they thought it could improve the hospital’s reputation.

As for clinical measures, the HbA1c tests were conducted by the MUST hospital lab at baseline and endpoint as part of our study agreement with the head of clinical pathology. Random blood glucose levels were measured using the “GlucoDr.” before, during‚ and after the SMS intervention. Recommended by our diabetes specialist, the “GlucoDr” was purchased particularly for our study due to its ease of use, small size, reasonable price, and widely available/affordable test strips. The device is also known for its high level of accuracy among this category of glucometers. One thousand test strips, lancets‚ and wiping pads were additionally purchased with the device and kept in the clinic. Weight measurements were performed with the clinic’s digital scale in increments of 100 g.

### Sample size

The primary hypothesis of the study was that intervention patients receiving daily educational SMS messages would experience a reduction in their HbA1c levels compared to controls given only paper-based instructions. Accordingly, sample size calculations were performed with the procedure “Two-sample t-tests (equal variances)” using nQuery 7.0, based on the results of a similar study by Kim et al. (2007) [38]. The effect of both the intervention and control groups on HbA1c at 3 months (−1.15 and 0.07 respectively) was used. Further, using the HbA1c standard deviations of both groups at 3 months (1.04 and 0.91), a common standard deviation of 0.976 was estimated. The error and power were set to 0.05 (two-sided) and 90% respectively. Calculations indicated that a sample of 80 patients (*n* = 40 per group) would be sufficient to detect significant changes in HbA1c after 3 months. Ten extra patients were recruited to account for dropouts if any. The biostatistical advice was provided by the Hannover Medical School (MHH) Institute for Biometry.

### Randomization and stratification

In order to prevent imbalance in prognostic factors, stratified randomization was implemented using the following stratification factors: age, sex, diabetes years, and SMS familiarity. Age and sex were considered of high importance in order to achieve balance within subgroups. Moreover, the proportion of patients that had been newly diagnosed or had only had diabetes for a short while as opposed to patients that already had experience with their diabetes was incorporated. Avoiding discrepancies in the number of patients that could read the SMS messages alone vs. those who needed someone to read for them was also considered necessary. This was based on the assumption that patients who were SMS familiar would likely be expecting the messages and would be curious to read them directly upon receipt. However, patients who relied on someone to read for them might be forced to wait or accumulate the messages until meeting that person, thus increasing the chance of missing or disregarding some messages. Randomization was additionally done by the MHH Institute for Biometry using the minimization algorithm. A patient list was submitted to them on March 5th and the randomized patient table was received on March 20th 2015.

### Ethics

There were no associated risks with our SMS intervention. The messages only reminded patients to follow their doctors’ instructions and did not interfere with their prescribed medications or insulin doses. For instance, sample messages included: "Do not forget to take your medications at the preset times" or "The medications will not be effective if not combined with regular exercise and healthy eating". The intervention aimed to provide an easier way to reach patients and educate them about their disease. It also intended to encourage self-management in order to help patients maintain a good state of health and avoid short and long-term complications. In that sense, meetings with the MUST ethics committee manager were initiated in June 2014, and verbal authorization to start recruiting patients was granted. Adjustments were made to the consent form explaining to patients the risks and benefits associated with the study, clarifying who they should contact in case they experienced any difficulties, and confirming their right to withdraw from the study at any point in time. To ensure conformity with the declaration of Helsinki, the study was approved by the MUST Ethical Research Committee (Protocol # 2014/3) on October 22nd 2014.

### Statistical analysis

Descriptive statistics were computed for baseline demographic and clinical characteristics; and reported as means and standard deviations for continuous variables, and frequency counts and percentages for categorical variables. Data not available or not applicable were coded as missing. Differences in secondary outcomes between groups at baseline and 12 weeks were examined using the independent samples t-test and the chi-square test for continuous and categorical variables respectively. The Fisher’s exact test was applied in cases where the chi-square test might not have been valid. An Analysis of Covariance (ANCOVA) was used to assess the change in HbA1c across study groups over 12 weeks, adjusting for baseline HbA1c values and incorporating the four stratification factors. The analysis was carried out using SPSS version 23, and the significance level and confidence interval were set to 0.05 and 95% respectively.

## Results

### Participant flow

**Fig. 1:**
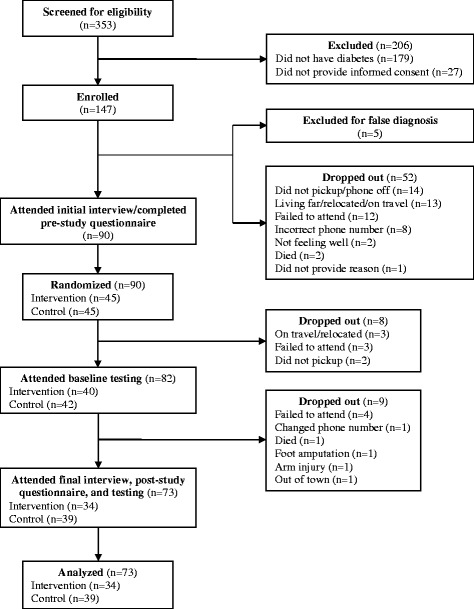
Recruitment Flowchart

Three hundred fifty three patients were assessed for eligibility during both the recruitment (April–October 2014) and replacement (November 2014–March 2015) periods. Two hundred six were excluded as 179 did not have diabetes and 27 did not sign the informed consent form. The remaining 147 patients were enrolled for the study. The recruitment flowchart is shown in Fig. 1.

Through the end of the baseline interviews, we excluded 5 more patients for being misdiagnosed with diabetes and observed a series of dropouts that caused the loss of 52 patients. Though a surprisingly high and unexpected dropout percentage at the time, the reasons were not due to loss of interest in the study. They were mainly because patients had to wait 3–6 months since initial recruitment to be contacted for the interviews, a period during which their living conditions and social circumstances had changed. Fourteen patients had their phones off or did not answer their phones despite several trials via calls and SMS messages; 13 indicated they were on travel, living too far, or had relocated to another city; 12 failed to attend their appointments; 8 mistook while writing their phone numbers at the time of enrollment; 2 indicated not feeling well due to surgery or pregnancy/illness and therefore could not make it to the interview; 2 passed away; and 1 did not give a reason. A total of 90 patients attended the baseline interview, completed the pre-study questionnaire, and were randomized to a control (*n* = 45) and an intervention (*n* = 45) group.

Between March 1st and March 15th 2015, 40 intervention and 42 control patients completed the HbA1c baseline tests and received the instruction booklet and the monitoring table. Of the 8 dropouts, 3 indicated they were on travel or had relocated to another city, 3 failed to attend their appointments, and 2 did not answer their phones. At endpoint, 6 intervention and 3 control patients were additionally lost and failed to attend the final HbA1c test. Of the 6 intervention patients, 4 failed to attend their appointments despite several calls and SMS messages, 1 changed their phone number, and 1 passed away. Of the 3 control patients, 1 had a foot amputation and could not come to the appointment, 1 had an arm injury, and 1 was out of town. A total of 34 intervention and 39 control patients attended the final interview, completed the post-study questionnaire, took the final HbA1c test, and returned their monitoring table. All 73 patients were included in our statistical analysis.

### Participant characteristics

Baseline demographic and clinical characteristics of patients that completed the study and were included in the analysis (*n* = 73) are shown in Table 1. Although not part of the inclusion criteria, all participants were Type 2 diabetics. For both groups, most patients were in their 50s and slightly over 50% were female. On the social level, most group participants were married, employed, educated enough to read Arabic, could open and read SMS messages on their own, and could afford buying their diabetes medications either at their own expense or with their health insurance plan. The intervention group had a somewhat higher percentage of employed participants with jobs on the university campus. Concerning medical history, a few patients from each group indicated suffering other chronic diseases such as hypertension, asthma, allergy, or heart or bone problems. The majority of group participants were already having some level of diabetes complications such as numbness in legs and feet (neuropathy), weak eye sight or cataract, heart disease, or kidney problems. Very few patients had received diabetes education as part of their nursing job in the hospital or at workshops held by MUST or Sukar Mazboot. The majority had had diabetes for more than a year‚ with an average of 5.63 and 7.99 years for control and intervention patients respectively.

**Table 1:** Participants’ baseline demographic and clinical characteristics (*n* = 73)

Characteristic	Control (*n* = 39)	Intervention (*n* = 34)
Personal:		
Age (years)	51.77 ± 9.68	51.24 ± 8.66
Sex (female)	23 (58.97)	18 (52.94)
Social:		
Married	32 (82.05)	30 (88.24)
Employed	23 (58.97)	27 (79.41)
University/hospital employee	13 (56.52)	19 (70.37)
Can read	28 (71.79)	28 (82.35)
SMS familiar	27 (69.23)	25 (73.53)
Can afford medications	32 (82.05)	33 (97.06)
Medical History:		
Diabetes years (≥1)	32 (82.05)	29 (85.29)
Type 2 diabetes	39 (100)	34 (100)
On insulin	7 (17.95)	7 (20.59)
Hypertensive	18 (46.15)	12 (35.29)
Other chronic diseases	12 (30.77)	10 (29.41)
Suffer diabetes complications	31 (79.49)	28 (82.35)
Suffered diabetic coma in last 3 months	5 (12.82)	5 (14.71)
Hospital/ER in last 3 months	5 (12.82)	4 (11.76)
Received diabetes education	2 (5.13)	4 (11.76)
Lifestyle & Monitoring:		
Smokers	6 (15.38)	10 (29.41)
Smokers wishing to quit	6 (100)	9 (90)
Average frequency of blood glucose measurement (SCI, 5)	2.28 ± 1.15	2.38 ± 0.95
Glucometer at home	12 (30.77)	15 (44.12)
Average rate of physical activity (SCI, 5)	3.79 ± 1.70	3.76 ± 1.60
Average rate of following a healthy diet (SCI, 5)	1.64 ± 1.18	1.76 ± 1.13
Average commitment to visiting diabetes doctor (SCI, 5)	1.59 ± 1.19	1.65 ± 1.25
Average rate of adherence to medication times (SCI, 5)	3.90 ± 1.21	3.94 ± 1.20
Perform daily feet check	27 (69.23)	26 (76.47)
Satisfied with body weight	16 (41.03)	16 (47.06)
Satisfied with own diabetes control	5 (12.82)	4 (11.76)
Satisfied with follow-up & communication with diabetes doctor (5)	2.27 ± 0.52	2.38 ± 0.52
Clinical:		
HbA1c (%)	9.53 ± 2.78	9.78 ± 2.53
Random blood glucose (mg/dl)	220 ± 103	242 ± 95
Weight (Kg)	89.9 ± 17.8	85.9 ± 13.8

Data are presented as Mean ± SD or Number of Patients (%)

In the lifestyle and monitoring category, all control and 90% of intervention group smokers indicated they wished to quit. Both group participants were very similar in their average rates of lifestyle behaviors such as frequency of blood glucose measurement, physical activity, following a healthy diet, visiting their doctors, and taking their medications on time. These behaviors were scored using the 5-point scale SCI, with 5 indicating the highest and most optimal rate and 1 indicating the lowest or non-existing rate. Only 6 control and 4 intervention patients indicated testing their blood glucose on a weekly basis, while 18 control and 13 intervention patients indicated walking a minimum of 30 min a day. Further, only 11 control and 9 intervention patients indicated having a regular schedule for seeing their diabetes doctor, while remaining patients only visited their doctors when not feeling well or not at all. Nearly half the participants of each group were comfortable with their body weight, while the majority of remaining patients wished to lose weight. Most participants had a low level of satisfaction with their diabetes doctor and very few were satisfied with their own diabetes control. Clinical factors such as HbA1c values, blood glucose levels, and body weight were similar among both groups.

### Main outcome findings

Over 12 weeks, a total of 3880 SMS messages were sent from “DiabAwPro”. Each intervention group patient received 97 messages, comprised of 84 educational and 12 reminder messages in addition to 1 welcome message. At baseline, no significant differences were observed between groups in main outcome measures or stratification factors (*p* = 0.587, 0.604, 0.709, and 0.686 for age, sex, diabetes years, and SMS familiarity respectively), thus indicating successful randomization. Our primary outcome, the change in HbA1c from baseline, did not differ significantly (Δ 0.290; 95% CI -0.402 to 0.983; *p* = 0.406) between groups after 3 months, demonstrating an unadjusted mean drop of −0.69% and −1.05% in the control and intervention group respectively. However, 16 intervention patients managed to achieve the targeted 1% drop as opposed to only 6 controls, suggesting a clear relationship between belonging to one of the study groups and accomplishing a 1% HbA1c drop (chi-square = 8.655; df = 1; *p* = 0.003).

All secondary outcomes appeared to be in favor of the intervention group after 3 months. Mean blood glucose levels decreased by 19 mg/dl among control patients and by 61 mg/dl among intervention patients. The average body weight showed a small drop of 0.5 Kg in the control group as opposed to 1.3 Kg in the intervention group. Treatment adherence improved slightly among control patients and considerably among intervention patients. No change in medication adherence was observed in the control group while a considerable improvement was noticed in the intervention group. Patients’ confidence in their ability to manage their disease slightly declined in the control group but notably improved among intervention patients. Compared to the patients’ level of confidence in their healthcare providers before the study, confidence in our program was substantially higher in both groups, with a slight advantage for intervention patients. Diabetes knowledge increased to a greater extent among intervention group patients compared to control patients.

From baseline to endpoint, 4 patients in the control group were admitted to the emergency room due to their diabetes as opposed to none in the intervention group. Both groups showed great improvement in their average frequency of blood glucose measurement, which was set to weekly as recommended by our diabetes doctor and scored 4 on the SCI scale. The number of patients that achieved the weekly check drastically increased, leaving only 5 intervention vs. 13 control patients not complying at 3 months. The average rate of regular activity slightly declined for the control group but considerably improved for the intervention group, with 13 additional intervention patients reporting walking at least 30 min a day as opposed to only 2 controls. Patients’ satisfaction with follow-up and communication with our program was remarkable for both groups at 3 months, highly exceeding their satisfaction scores with their healthcare providers before the study. All participants indicated high level of satisfaction with the program, expressed interest to remain in the program should it continue to operate, said they would recommend it to others, and believed it could improve the hospital’s reputation. The main findings of the study are detailed in Table 2.

**Table 2:** Intervention effects on primary and secondary outcomes, self-management behaviors, and education items

Outcome		BASELINE			ENDPOINT (3 MONTHS)		
		Control (*n* = 39)	Intervention (*n* = 34)	*P*	Control (*n* = 39)	Intervention (*n* = 34)	*P*
Primary Outcome							
HbA1c (%)		9.53 ± 2.78	9.78 ± 2.53	0.690	8.84 ± 2.40	8.73 ± 1.98	0.838
Change in HbA1c from baseline	Unadjusted mean	–	–	–	−0.696	−1.053	0.454
	Adjusted mean				−1.389	−1.679	0.406
Patients that achieved at least 1% HbA1c drop		–	–	–	6 (15.38)	16 (47.06)	0.003
Secondary Outcomes							
Random blood glucose (mg/dl)		220 ± 103	242 ± 95	0.353	201 ± 87	181 ± 65	0.288
Weight (Kg)		89.9 ± 17.8	85.9 ± 13.8	0.287	89.4 ± 18.1	84.6 ± 14	0.215
Treatment adherence (SCI), (5)		2.23 ± 0.51	2.23 ± 0.53	0.995	2.52 ± 0.49	3.42 ± 0.48	<0.0001
Medication adherence (Morisky), (4)		2.74 ± 0.99	2.74 ± 1.19	0.974	2.74 ± 1.07	3.76 ± 0.55	<0.0001
Diabetes self-efficacy (patient confidence), (5)		2.82 ± 0.41	2.88 ± 0.43	0.576	2.68 ± 0.33	3.51 ± 0.39	<0.0001
Patients’ confidence in HP/program, (5)		2.44 ± 0.72	2.59 ± 0.74	0.377	4.03 ± 0.28	4.21 ± 0.48	0.059
Diabetes knowledge, (1)		0.29 ± 0.20	0.35 ± 0.23	0.278	0.34 ± 0.21	0.73 ± 0.18	<0.0001
Patients not admitted to ER/hospital in last 3 months		34 (87.18)	30 (88.24)	1.000	35 (89.74)	34 (100)	0.118
Average frequency of blood glucose measurement, (5)		2.28 ± 1.15	2.38 ± 0.95	0.688	3.21 ± 1.28	3.88 ± 0.77	0.007
Patients that checked blood glucose at least once a week		6 (15.38)	4 (11.76)	0.742	26 (66.67)	29 (85.29)	0.065
Exercise (patients that walked at least 30 mins/day)		18 (46.15)	13 (38.24)	0.495	20 (51.28)	26 (76.47)	0.026
Average rate of regular activity, (5)		3.79 ± 1.70	3.76 ± 1.60	0.938	3.77 ± 1.75	4.74 ± 0.86	0.004
Patient satisfaction with follow-up & communication with HP/program, (5)		2.27 ± 0.52	2.38 ± 0.52	0.360	4.03 ± 0.28	4.21 ± 0.48	0.059
General patient satisfaction with program		100% of patients indicated general satisfaction with the program, said they would stay enrolled should the program continue, and said they would also recommend it to others.					
Healthcare provider’s reputation		100% of patients believed the program could improve the hospital’s reputation.					
Other Self-management behaviors							
Smokers/patients that reduced or stopped smoking		6(15.38)	10(29.41)	0.148	3 (50)	8 (80)	0.299
Patient commitment to seeing their doctors at preset times, (5)		1.59 ± 1.19	1.65 ± 1.25	0.842	1.33 ± 0.93	2.26 ± 1.67	0.006
Results recording, (5)		1.38 ± 1.02	1.41 ± 1.16	0.915	2.90 ± 1.94	4.09 ± 1.42	0.004
Adherence to medication times, (5)		3.90 ± 1.21	3.94 ± 1.20	0.878	3.90 ± 1.23	4.76 ± 0.78	0.001
Adherence to insulin dose adjustment, (5)		1.14 ± 0.38	1.50 ± 0.84	0.368	1	2 ± 1.53	0.134
Adherence to follow-up tests, (5)		1.18 ± 0.68	1.21 ± 0.64	0.866	1.15 ± 0.67	1.74 ± 1.05	0.008
Patients that performed daily foot check and care		27(69.23)	26(76.47)	0.489	31(79.49)	34(100)	0.006
Patients satisfied with their diabetes control		5(12.82)	4(11.76)	1.000	8(20.51)	14(41.18)	0.055
Adherence to following healthy diet, (5)		1.64 ± 1.18	1.76 ± 1.13	0.650	2.31 ± 1.62	3.35 ± 1.57	0.007
Adherence to carrying a strong-acting sugar, (5)		2.10 ± 1.62	2.41 ± 1.84	0.448	2.41 ± 1.80	2.97 ± 1.88	0.199
Confidence in ability to know when it is necessary to see the doctor, (5)		2.51 ± 0.64	2.53 ± 0.79	0.921	2.26 ± 0.50	3.06 ± 0.81	<0.0001
Education Items							
Patients aware of side effects of their medications		2(5.13)	2(5.88)	1.000	1(2.56)	14(41.18)	<0.0001
Patients aware of diabetes complications		13(33.33)	16(47.06)	0.232	17(43.59)	32(94.12)	<0.0001
Patients aware of follow-up tests		4(10.26)	6(17.65)	0.499	5(12.82)	28(82.35)	<0.0001
Patients aware of healthy diet		15(38.46)	16(47.06)	0.459	24(61.54)	34(100)	<0.0001
Patients aware of physical activity benefits		18(46.15)	16(47.06)	0.938	22(56.41)	32(94.12)	0.0002

Data are presented as Mean ± SD or Number of Patients (%) unless otherwise specified; *ER* Emergency room, *HP* Healthcare provider

## Discussion

### Related work

This study examined the feasibility of a diabetes educational SMS program targeting glycemic control and self-management behaviors among Egyptian diabetics. Based on the opinion of our local doctors, patients needed to undertake healthy eating, regular physical exercise, and high rates of medication adherence in order to achieve better glycemic control. However, eating healthy, being physically active, and taking medications on time are only a subset of various self-management behaviors that diabetic patients need to maintain. Others include regular blood glucose monitoring, results recording, regular visits to diabetes doctor, commitment to diabetes follow-up tests, regular foot-checking, knowing what to do in cases of hypo- or hyperglycemia, knowing when it is necessary to see the doctor, quitting or reducing smoking, adjusting insulin dosage based on blood glucose readings and food intake, and always carrying a strong-acting sugar. At the time of preparation for this study, the authors were not able to identify other studies that addressed all these factors together. Studies usually focused on one or some of these behaviors or on glycemic control and its associated behaviors.

Our study contributes to the increasing mHealth body of literature suggesting that SMS interventions are feasible tools for diabetes management with great potential to improve clinical outcomes. In a study in the Netherlands that used SMS reminders to improve medication adherence over a period of 6 months, intervention patients took significantly more medication doses than control patients (50% vs. 39% within a 1-h window and 81% vs. 70% within a 4-h window) and had a 5% lower rate of missing their doses [39]. In another study in the US, SMS messages were sent to a group of 18 patients for 1 month, addressing medication adherence and self-management behaviors such as foot checking and blood glucose monitoring. Missed medication doses significantly decreased from 1.6 to 0.6 per week, and diabetes self-efficacy significantly increased during the study (*p* = 0.002). Further, 89% of patients indicated increased frequency of foot self-examinations [40].

In Iran, a study showed that SMS messages were as effective as telephone calls in monitoring 77 Type 2 diabetic patients. SMS group patients (*n* = 38) received 4 messages per week on diet, exercise, medication taking, and frequent self-monitoring of blood glucose levels; and achieved 1.01% drop in the mean HbA1c level at 3 months. Telephone group patients (*n* = 39) achieved almost the same result (0.93% drop), yet the calls required more time and money than the SMS messages. No results were reported on diet, exercise, medications‚ or other self-management behaviors [41]. A study in South Korea asked participants to enter their blood glucose levels, diet, and exercise diaries into a website on a daily basis, and accordingly sent them weekly SMS recommendations. Compared to baseline, the mean HbA1c level decreased by 1.15% at 3 months and 1.05% at 6 months for intervention patients (*n* = 25), and increased for control patients (*n* = 26) by 0.07% and 0.11% at 3 and 6 months respectively. Fasting plasma and 2 h post-meal glucose levels were also recorded (with intervention patients achieving significant declines by 85.1 mg/dl and 63.1 mg/dl at 3 and 6 months respectively) but no self-management behaviors were monitored [38].

In the SuperEgo study, 23 Type 1 diabetes adolescents received tailored SMS messages at an average of 10 messages/week based on their individually reported diabetes self-care. The messages addressed stress, exercise, communication, social support and stigma, time planning, reminders, and dietary portions. At 3 months, intervention patients maintained their mean HbA1c baseline levels while control patients significantly worsened (*p* = 0.006). Only usability and satisfaction were additionally evaluated but no self-management behaviors were addressed [42]. In the Sweet Talk study, that also aimed to improve glycemic control among Type 1 diabetics, intervention participants received daily SMS messages on insulin injections, blood glucose testing, healthy eating, and exercise. Over 12 months, the mean HbA1c levels did not change significantly in control or intervention patients. Yet, diabetes self-efficacy as well as other self-management behaviors such as blood glucose testing, healthy eating, and exercise (all measured by a diabetes social support interview) were significantly better in the intervention group at the end of the study [43].

A study in Bahrain examined the effect of bidirectional SMS messages on participants’ glycemic control. Patients sent their inquiries about diet, medications, side effects, blood glucose levels, hypo- and hyperglycemia actions, and follow-up tests; and received immediate feedback from the medical doctor. Patients that did not send any inquiries for 7 consecutive days were sent SMS reminders. After 3 months, both intervention (*n* = 12) and control (*n* = 22) patients achieved significant declines in their HbA1c levels (−2.76% and −1.6% respectively) compared to baseline. Intervention patients, however, achieved a significantly higher reduction in their mean HbA1c, 1.16% points lower than that of controls. No results on self-management behaviors were reported [44]. Another study in South Korea asked participants to enter their blood glucose levels daily into a website and sent them recommendations by SMS accordingly. After 6 months, the intervention group (*n* = 18) had a statistically significant decrease in HbA1c, fasting plasma glucose, and 2-h post-meal glucose levels compared to the control group (*n* = 16). A significant mean percentage change of −1.22 and −1.09 was recorded at 3 and 6 months respectively for the intervention group, while the control group did not show any significant changes. No self-measurement behaviors were measured [45].

In Norway, the impact of using the Few Touch Application (FTA) on self-management was examined. Participants were assigned either to an FTA (*n* = 51), FTA and phone health counseling (HC, *n* = 50), or a control (*n* = 50) group. The FTA consisted of a blood glucose measuring system, a diet manual, and a physical activity diary. After 1 year, the primary outcome (HbA1c) decreased in all groups but did not significantly differ between groups. Secondary outcomes including weight, depressive symptoms, and nutritional and exercise habits also did not change significantly between groups after 1 year [46].

The CareSmarts study in Chicago evaluated the impact of bidirectional SMS messages on HbA1c levels and self-management behaviors such as healthy eating, exercise, foot checking, and blood glucose testing over 6 months. Participants of the intervention group achieved a significant drop in their HbA1c levels (7.9 to 7.2%), and significantly improved their frequency of healthy eating, blood glucose monitoring, and performing foot checks. Medication adherence also significantly improved at the end of the study. No changes in clinical outcomes were observed in control group patients though and the main limitation was lack of randomization [47]. Another study in the US assigned its participants to a text messaging group that received daily messages on nutrition and physical activity, and a control group that received a pamphlet on healthy lifestyle. The 1-month trial was too short to see significant changes in HbA1c levels, self-efficacy, or body mass index for any of its groups. However, satisfaction with the SMS messages was high [48].

In the TExtT-MED study, 128 patients were randomized to an intervention group (*n* = 64), that received 2 daily text messages in English or Spanish, and a control group (n = 64). The primary outcome was the change in the median HbA1c level. Secondary outcomes included changes in medication adherence, self-efficacy, diabetes knowledge, emergency department (ED) utilization, self-care tasks such as foot checking and blood glucose measurement, and patient satisfaction. After 6 months, the median HbA1c decreased by 1.05% in the intervention group as opposed to 0.6% in the control group, a change that was not considered significant. However, secondary outcomes showed considerable improvement particularly in medication adherence and ED utilization. The study’s restricted focus on ED patients was found to limit the generalizability of its findings [49].

### Trends of improvement

In our study, the change in HbA1c from baseline did not differ significantly between groups after 3 months, yet a significantly higher number of intervention patients managed to achieve a 1% drop in their HbA1c levels. Trends of improvement were also observed in secondary outcomes, self-management behaviors, and education aspects. The reasons why particularly 16 intervention patients could achieve the 1% drop while the remaining 18 patients could not were not very clear. However, exploring the baseline characteristics of these patients brought some interesting facts to our attention. The most noticeable differences between achievers and non-achievers were in their baseline HbA1c values, sex, SMS familiarity, confidence in healthcare provider, and satisfaction with own body weight.

All achievers had baseline HbA1c levels above 8%, ranging from 8.2% to 15.4%. Non-achievers had a lower range (5.8 to 13.5%) and 9 of them had values already below 8%. Most male intervention patients were achievers (*n* = 11), while most females were non-achievers (*n* = 13). Of the 9 intervention patients that were not SMS familiar and had someone read the messages for them, only 2 achieved the drop while 7 were non-achievers. The majority of patients that indicated having low confidence in their healthcare providers were achievers (*n* = 12), while the majority that had higher levels of confidence were non-achievers (n = 11). Finally, most patients that indicated satisfaction with their body weight managed to achieve the drop (n = 12), whereas the majority of patients that were uncomfortable with their body weight were non-achievers (*n* = 14). Age, years of diabetes, social status, among other factors did not notably differ between achievers and non-achievers. However, most of the patients who achieved the drop were surprisingly aged over 50.

We likely have to differentiate between having good glycemic control and the practice of self-management behaviors. While eating, exercising, and medication taking behaviors are the ones that could have a direct impact on HbA1c levels, a person with good glycemic control is not necessarily one with good self-management and vice versa. Since studies mostly focus on one aspect or the other, this relationship is rarely observed [46]. One of the main advantages of this study is that it measured several factors besides HbA1c and blood glucose levels, such as treatment and medication adherence, diabetes self-efficacy, and diabetes knowledge. Further, not only did it incorporate self-management behaviors within such factors or address them in the SMS categories, but it also produced individual scores for these behaviors to measure improvement. As both study groups were presented with an intervention (booklet vs. booklet + SMS), it should not be surprising to see progress in both groups. However, we were expecting greater improvement among intervention patients who were constantly reminded and motivated by SMS. Therefore, the effect of SMS can be seen by examining the difference in levels of improvement between groups rather than looking for improvement in the intervention group vs. no/slight changes in the control group.

Though the HbA1c levels did not differ significantly between groups at 3 months, considerable differences were observed in many of the secondary outcomes and the self-management behaviors (Table 2). All intervention patients performed daily foot checks during the study period (8 additional to baseline) vs. 79.45% of control patients (only 4 additional to baseline). Of participants who indicated suffering extreme increase or decrease in their blood glucose levels at 3 months, 13 control patients indicated they did not know which action to take as opposed to only 2 intervention patients. Patients’ ability to know when they have to see their diabetes doctor considerably differed between groups at the end of the study (scoring 2.26 vs. 3.05 on DES-SF), slightly dropping from its baseline score for controls and improving for intervention patients.

The mean rates of recording blood glucose measurements, visiting the diabetes doctor at preset times, and adhering to follow-up tests were poor for both groups at baseline, but became considerably higher for intervention patients at 3 months. The rate of adjusting the insulin dose based on food intake remained low for both groups at the end of the study, yet slightly improved for intervention patients and slightly declined for controls compared to baseline. Rate of carrying a strong-acting sugar somewhat improved for both groups but remained slightly higher for intervention patients at 3 months. Very few patients in both groups indicated satisfaction with own diabetes control at baseline. However, 41.18% of intervention patients vs. 20.51% of controls indicated satisfaction at endpoint. The HbA1c associated behaviors such as rate of following a healthy diet, being physically active, and adhering to prescribed medications produced remarkably higher scores for intervention patients at 3 months.

### Possible causes for control group improvements

Our results appear to be in line with previous studies that mostly did not detect significant differences in HbA1c within 3, 6, and even 12 months; yet observed significant improvement in secondary outcomes when they existed. It is important to note, however, that studies that did find significance in HbA1c usually did not provide their control groups with any types of interventions (only usual care), thus achieving significance through improvement of their intervention group over non-changing or worsening control group. Further, the HbA1c improvement of such studies usually fell in the range of −1% within 3 to 6 months, a drop that was already achieved by our study’s intervention group after 3 months. Therefore, in order to explain why we could not achieve significance in HbA1c, we need to look into causes for improvement in our control group rather than barriers to greater improvement in the intervention group. The first cause, as mentioned earlier, is clearly that the control group was also provided with a form of intervention. Upon getting the instruction booklet, patients were expected to read it then put it aside for the rest of the study period or not read at all. However, intervention patients were constantly reminded by the instructions through regular SMS messages, and therefore they were expected to achieve significantly greater improvement. At endpoint, 20 controls vs. 30 intervention patients indicated reading the instruction booklet. We believe that significant differences could have been detected had we not given an instruction booklet to either groups or monitored them via a monitoring table.

The second cause maybe the monitoring table that controls were given and that also represented a form of intervention. Having this table and knowing that it would be reviewed in 3 months likely encouraged patients to check their blood glucose levels regularly and fill in their results. Further, looking at their recorded results possibly pushed them to adhere to treatment in order for the next measurement to show improvement. Between baseline and endpoint, the results recording mean SCI score increased from 1.38 to 2.90 for controls, and from 1.41 to 4.09 for the intervention group. This result was expected for intervention patients who were reminded on a weekly basis by SMS to check and record, but it was surprising for controls who were expected to also place the monitoring table aside upon receiving it, and forget to check and record every week.

The third reason may be the short period of the study. Given the poor communication our patients had with their doctors, sitting through the 30-min baseline interview, getting a chance to express their concerns about diabetes, feeling that they were being monitored and that they belonged to a program, and knowing that they would come back for another interview in 3 months were all sufficient reasons to keep all our patients motivated. Since the effect of our SMS intervention relied on sustained motivation, a period of 3 months was probably too short for the levels of motivation to decline given the above circumstances. However, had the study extended to a longer period, we would have expected the motivation of controls to drop while that of interventions to be maintained by receiving the daily SMS messages.

The fourth reason may be that many of our study participants knew each other. At the time of enrollment, upon learning about the benefits of the study, participants brought their diabetic family members and friends to also participate. Further, 13 controls and 19 intervention patients were already working together on the university campus, either in the hospital, administrative departments, or in one of the faculties. Therefore, it is highly likely that there was contact between members of the intervention and the control groups throughout the course of the study, particularly among those who worked in the same buildings (e.g. nurses, housekeeping, etc.). Consequently, the motivation that the SMS messages brought to intervention patients might have been transferred to some control patients leading to unexpected improvement.

### SMS vs. traditional methods

Besides glycemic control and self-management, our study aimed to also address patient education. The SMS intervention remarkably improved knowledge aspects (Table 2) and showed high levels of acceptance over traditional education methods such as paper-based materials, lectures‚ or seminars. In general, designing and printing booklets or pamphlets require time and money, and patients are usually discouraged to take the time to read and process their load of information at once. Likewise, the organization of lectures and seminars requires costly efforts, and patients face the barriers of distance and time to attend them. In-clinic education during medical visits depends primarily on patients’ commitment to see their doctors regularly, and on doctors providing the time to have discussions with their patients. Further, patients are likely to forget or start ignoring their doctor’s tips a few days after the visit, or lose motivation to comply with treatment [50]. In light of such circumstances, SMS messages present a low cost method that can deliver educational material to patients wherever they are. They do not require complicated smartphones and can be easily checked and read without any distance or time barriers. If sent regularly, they can also provide light information doses that can easily be read and understood at once, and sustain patients’ motivation to adhere to treatment.

In our study, given the poor levels of communication with healthcare providers, most patients were not receiving the most basic form of education in the clinic. Insured patients indicated they were given their medication doses every 2–3 months without even being checked by a doctor, and accordingly knew very little about their diabetes and how to control it. Very few patients indicated attending educational lectures or seminars (2 controls to 4 interventions) which were rarely organized. One of the notable advantages of our SMS intervention was the motivation it brought to patients to read the more detailed paper-based instructions, as only 51.28% of control patients indicated reading the booklet vs. 88.24% of intervention patients. Further, 55.88% of intervention patients preferred the SMS messages to the booklet, 20.59% said they liked having them both, while only 23.53% favored the booklet. Reasons for favoring SMS messages included their continuity (daily rate); their reminding nature; being short, simple and quick to read; mobile phone being always within reach; and the likelihood of being discouraged to read the booklet, losing it, or forgetting about it.

Four of 8 patients who preferred the booklet were not familiar with SMS. They indicated that it would be easier and less awkward to ask someone to read the booklet for them once rather than daily SMS messages. The other four preferred the booklet since it contained information that they did not have to wait for, it was always there but they sometimes had to delete the SMS messages, they did not have to look for the information in their phones, and they were concerned about not getting the SMS. Patients that preferred having both the booklet and SMS messages together indicated they integrated each other, since the booklet would be there to keep and refer to any time, and the SMS messages would act as daily quick reminders.

It was encouraging to see that patients’ opinion of our program was generally positive, and that satisfaction and acceptance of SMS messaging as a method of communication, education, and sustaining motivation was high. Of the 34 intervention patients, 27 indicated receiving and reading the SMS messages every day, one patient indicated missing a few as a result of spending some days in places with poor network coverage, and 6 indicated missing the majority of messages for the following reasons: 3 did not pay much attention to their phones, 2 indicated the person that was supposed to read for them was not available every day, and one patient often left their phone with someone else. All patients that got the SMS messages (*n* = 28) found no difficulty reading or understanding them and thought no information was missing in their content. Moreover, they appreciated the daily rate of SMS sending and thought the morning receipt times were appropriate. When our study participants (*n* = 73) were asked if they felt something was missing in the program, 21 indicated the following: Some wished we had provided more incentives such as free medication doses, glucometers, or additional lab tests; others wished our clinic’s doctor had changed their medication doses or prescribed them new ones; and a few indicated they did not know they could visit the clinic’s doctor free of charge during the time of the study and wished they had been examined.

### Limitations

The most obvious limitation in our study is its short period. We believe that 6 months is the optimal timeframe for studies looking for changes in glycemic control. However, due to budgetary constraints in addition to the increased risk of losing patients through longer periods, we restricted our study to 3 months. The study’s small sample size may also limit generalizibility, thus needing a larger RCT to establish clinical efficacy/effectiveness. Time delays during recruitment and overlaps between study phases could also be viewed as a limitation of the study. Unusual delays in ethical committee procedures resulted in prolonged waiting times between patient enrollment and beginning of baseline interviews. Offering incentives during this period could have minimized early dropouts and avoided initiation of a replacement phase. However, this was also not possible due to budgetary constraints. Baseline interviews and HbA1c tests were scheduled to take place at one appointment. Yet, as patient randomization was still in process, we postponed the collection of HbA1c samples to avoid large time gaps between baseline testing and start of intervention. On the other hand, it was necessary to start contacting enrolled patients and ask them to come back in order to avoid further dropouts. Therefore, splitting the baseline appointment into two meetings, one for the interview and pre-study questionnaire and another for the HbA1c samples, was highly recommended.

Given the amount of paperwork, approvals‚ and signatures that were needed from university administration, hospital management, faculty members, or participating team members through the different stages of the study, following a strict time plan was not always possible. Consequently, the delays in all study phases led to a 2-week overlap of the study period with the month of Ramadan, which forced us to start our final interviews 2 weeks ahead of schedule. Though this might not have had a noticeable impact on our results, it could still be seen as a limitation of our study. Lack of incentives could also be seen as a limitation. Due to budgetary constraints, we could only provide the HbA1c tests and visits to the clinic’s doctor free of charge. We believe, however, that other incentives such as free medication doses, glucometers, phone credit, or other lab tests could have minimized the dropout rate. Limiting our selection criteria to residents of October city could have also minimized patient dropout. Further, selecting only patients that had no contact with each other or patients that were SMS familiar could have produced better results. We chose, however, to keep our criteria unrestricted in order to attract more patients, especially that those that visit MUST hospital public clinics are usually not very well educated, and accordingly not good readers of SMS messages.

### Suggestions for future trials

Though our study followed an RCT methodology, seeking to assess the impact of the SMS educational intervention on health outcomes, its sample size and duration may not have been sufficient to indicate clinical efficacy/effectiveness. The study, however, establishes guidelines for future larger and longer RCTs targeting changes in HbA1c and self-management behaviors. For instance, a longer study is recommended to last at least 6 months in order to see significant differences in HbA1c, and preferably go to a year to allow for changes in behaviors to be observed. Further, stratifying patients into ones with HbA1c values of 8% or lower as opposed to those with higher values at baseline would be highly recommended, as such patients responded differently to our SMS intervention. In our short study, it was not possible to thoroughly evaluate rarely addressed behaviors such as visiting the diabetes doctor regularly or adhering to follow-up tests. This is because patients are generally advised to visit their doctors every 3 months, take a kidney function and a urine test every 6 months, and have an eye exam and a lipid profile test once a year (personal communication with Dr. Mohamed Alaa^4^). Other behaviors such as following a healthy meal plan and abstaining from smoking also need a longer time frame to be better evaluated.

Future studies are additionally advised to avoid overlapping with the month of Ramadan due to the effects of the special lifestyle of the month on diabetes, which could make it difficult to assess the impact of the SMS intervention independently. However, this could be challenging for studies aiming to last for 1 year, thus they are recommended to start shortly after Ramadan to avoid extended overlaps. Due to its short period, our study could only compare patients’ satisfaction with their healthcare providers before joining our program to their satisfaction with the program. A longer study could perform a more accurate comparison by adding an essential message category addressing the doctor-patient relationship; educating patients on how to maintain a good relationship with their doctors, what to do or inquire about at their medical appointments, and what to expect from their doctors; then finally compare their satisfaction with their healthcare providers before and after receiving such education. A message category on calculating insulin dosage and adjusting it based on food intake should also be incorporated, as this was not commonly known or done by insulin users in our study.

## Conclusions

SMS education appears to be a feasible and acceptable method for improving glycemic control and self-management behaviors among Egyptian diabetics. Yet, whether it is more effective than traditional paper-based materials remains a topic for further research. In our study, SMS messages resulted in higher HbA1c reductions than an instruction booklet after 3 months, but the most sizeable improvements were observed in secondary outcomes (treatment and medication adherence, diabetes knowledge, etc.) and self-management behaviors. Further, SMS messages were preferred to traditional methods in educating patients about their diabetes and sustaining their motivation to adhere to treatment.

In regards to glycemic control, our findings suggest that male patients in Egypt may be more interested in mobile technology interventions than females. Moreover, patients who require someone to read the messages for them may not benefit from SMS interventions as much as patients who are familiar with using mobile phones. Accordingly, future studies should look into ways to make mobile interventions more attractive to females, and train their participants to use the mobile phone’s features before starting the intervention. For patients who cannot read at all, SMS interventions may not be suitable. They may also be of limited effects to patients who are normally careless about their health and body shape, or patients that have moderate to high levels of confidence in their healthcare providers. In that sense, future studies should also explore ways to extra motivate patients that usually do not care for their health (message frequency, format, etc.) in order to encourage them to care for the SMS messages. Further, studies should clarify to participants that SMS interventions do not aim to replace personal communication with their doctors. SMS interventions appear to bring more benefit to diabetics whose HbA1c levels are higher than 8%, and could surprisingly attract older people in Egypt who are usually not very good users of mobile phones.

The findings of our study add to the increasing body of literature supporting mHealth as an innovative public health solution for people with diabetes. Improvement in HbA1c levels and self-management behaviors, along with the high level of satisfaction with the program and strong acceptance of SMS messages, are all factors that establish feasibility and acceptability of our SMS intervention among diabetics in Egypt. Yet, larger and longer RCTs are still needed to assess its clinical efficacy/effectiveness and guarantee the generalizability of our findings.

## Additional files

Additional file 1: Study questionnaires; pre- and post-study questionnaires with English translation. (PDF 283 kb)

Additional file 2: Instruction booklet and nutrition leaflet; both the instruction booklet and nutrition leaflet as given to patients followed by their English translation. (PDF 860 kb)

Additional file 3: SMS content; the educational SMS messages listed by category and followed by their English translation. (PDF 121 kb)

## Abbreviations

ADA: American Diabetes Association
ADSL: Asymmetric digital subscriber line
ANCOVA: Analysis of covariance
DES-SF: Diabetes empowerment scale-Short form
DiabAwPro: Diabetes awareness program
DKN: Diabetes knowledge
DSE: Diabetes self-efficacy
ED: Emergency department
EMR: Eastern Mediterranean Region
ER: Emergency room
FTA: Few touch application
HbA1c: Glycated hemoglobin
HP: Healthcare provider
IDF: International Diabetes Federation
ITU: International Telecommunication Union
LMICs: Low and middle income countries
MCIT: Ministry of Communications and Information Technology
mDiabetes: mobile diabetes
MHH: Hannover Medical School
MMAS-4: Morisky medication adherence scale
MOH: Ministry of Health
MOHE: Ministry of Higher Education
MUST: Misr University for Science & Technology
NCDs: Non-communicable diseases
RCT: Randomized controlled trial
SCI: Self-care inventory
SMS: Short message service
SPSS: Statistical Package for the Social Sciences
US: United States
WHO: World Health Organization

## Footnotes

1 Prof. Mahi Al Tehewy is the Director of the healthcare quality unit at Ain Shams University and consultant to the Egyptian Ministry of Health.

2 Dr. Amira El Ansary is an internal medicine specialist at MUST hospital.

3 Dr. Hani Farouk is the eHealth focal point at WHO EMRO.

4 Dr. Mohamed Alaa is an internal medicine doctor at MUST hospital.
