# Supplementary material for: SMS education for the promotion of diabetes self-management in low & middle income countries: a pilot randomized controlled trial in Egypt
Source: BMC Public Health. 2017 Dec 19;17:962. doi: 10.1186/s12889-017-4973-5 (PMC5735794; doi:10.1186/s12889-017-4973-5)
Supplement: Supplementary file 1 — Study questionnaires; pre- and post-study questionnaires with English translation. (PDF 283 kb) [file 12889_2017_4973_MOESM1_ESM.pdf]

برنامج التوعية و المتابعة لمرضي السكر

Diabetes Awareness Program
Pre-study Questionnaire

(General Information):معلومات عامة

Name
١. الاسم: ....

Participant Number
٢. رقم المشترك: ....

Body weight and random blood glucose level
٣. الوزن و السكر العشوائي: ....

Do you smoke? **Yes, at a rate of:** **No**
٤. هل أنت مدخن؟ ☐ نعم، بمعدل: .... ☐ لا

Do you wish to quit smoking? **Yes** **No**
٥. هل ترغب في التوقف عن التدخين؟ ☐ نعم ☐ لا

(Medical History):التاريخ الطبي

Are you hypertensive? **Yes** **No**
٦. هل أنت مريض ضغط؟ ☐ نعم ☐ لا

Other chronic diseases you suffer form:
٧. أمراض مزمنة أخري تعاني منها: ....

Other medications you regularly take:
٨. أدوية أخري تتناولها باستمرار: ....

How often do you visit the hospital's outpatients?
٩. كم مرة تتردد علي عيادات المستشفي؟☐أول مرة ☐اسبوعيا: ...... مرات ☐شهريا: ...... مرات ☐عند اللزوم
first time **weekly** **monthly** **Upon need**

(Diabetes History):تاريخ السكر

Current diabetes medications and medication times:
١٠. أدوية السكر في الوقت الحالي و مواعيدها : ....

Insulin: **Yes** **No**
١١. انسولين: ☐ نعم ☐ لا

Who do you see for your diabetes?
١٢. مع من تتابع مرض السكر؟ ☐ أخصائي سكر ☐ باطني
diabetes specialist **GP**

Where? hospital's outpatients Other clinics

١٣. أين تتابع مرض السكر؟ ☐ بعيادات المستشفي ☐ بعيادات أخري

How often do you see your diabetes doctor?

١٤. كم مرة تزور الطبيب بسبب السكر؟ ☐ يوميا: ...... مرات ☐ اسبوعيا: ...... مرات ☐ شهريا: ...... مرات ☐ عند اللزوم

Daily weekly monthly upon need

Do you commit to seeing your doctor at preset times? [1]

١٥. هل تلتزم بزيارة الطبيب [1] في المواعيد المحددة؟ ☐ دائما ☐ غالبا ☐ ٥٠ % من الوقت ☐ أحيانا ☐ لا

Always Usually 50% of time Sometimes No

In the last 3 months, how many times were you admitted to the ER or hospital because of your diabetes? [2]

١٦. كم مرة قمت بزيارة غرفة الطوارئ (أو تم احتجازك بالمستشفي) بسبب السكر في الثلاث أشهر الماضية؟ [2] .... مرات

How often do you check your blood sugar? [1, 3]

١٧. كم مرة تقوم بقياس [1, 3] السكر؟ ☐ يوميا: ...... مرات ☐ اسبوعيا: ...... مرات ☐ شهريا: ...... مرات ☐عند اللزوم ☐لا أقيس

daily weekly monthly upon need Do not check

Where do you check your blood sugar?

١٨. أين تقوم بقياس السكر؟ ☐ بالصيدلية ☐ بالمعمل ☐ يوجد جهاز قياس بالبيت

Pharmacy Lab Glucometer at home

Do you record your results? [1]

١٩. هل تقوم بتسجيل نتائج القياس؟ [1] ☐ دائما ☐ غالبا ☐ ٥٠ % من الوقت ☐ أحيانا ☐لا

Always Usually 50% of time Sometimes No

Why don't you check regularly?

٢٠. لماذا لا تقيس السكر باستمرار؟ ☐ لا يوجد تشجيع من العائلة ☐ أكره شك نفسي ☐ سعر التحليل
☐ مشغول أو خارج البيت ☐لا تشعر بتحسن ☐عندما أشعر بالاكتئاب أو الملل
☐ أسباب أخري ....

No support from family I hate pricking myself Test cost
busy/away from home I don't feel better Bored or depressed
Other

Usual test result:

٢١. النتيجة المعتادة لتحليل السكر: صائم (قبل الأكل) .... فاطر (بعد الأكل بساعتين) .... عشوائي ....

fasting 2-hrs postprandial Random

Have you recently taken the HbA1c test? Date Result

٢٢. هل قمت مؤخرا بعمل تحليل الهيموجلوبين السكري؟ .... التاريخ: .... النتيجة: ....

Do you take your medications/insulin on time? [1]

٢٣. هل أنت منتظم في مواعيد [1] الدواء أو الانسولين؟

☐ دائما **Always** ☐ غالبا **Usually** ☐ ٥٠ % من الوقت **50% of time** ☐ أحيانا **Sometimes** ☐ لا **No**

Excluding times you forget, are you sometimes careless taking your medicine/insulin? [4]

٢٤. عدا النسيان، هل تهمل أحيانا في تناول الدواء أو الانسولين؟ [4] ☐ نعم **Yes** ☐ لا **No**

At what rate do you miss taking your medications on time?

٢٥. بأي معدل تكون غير منتظم في تناول الدواء في مواعيده؟

☐ يوميا: ...... مرات **daily** ☐ اسبوعيا: ...... مرات **weekly** ☐ شهريا: ...... مرات **Monthly**

What makes you miss taking your medications on time?

٢٦. ما الذي يجعلك غير منتظم؟

☐ أعراض جانبية **side effects**
☐ لاتشعر بتحسن **I don't feel better**
☐ مشغول أو خارج البيت **Busy/away from home**
☐ لا يوجد تشجيع من العائلة **no support from family**
☐ سعر الدواء **price of medication**
☐ عندما أشعر بالاكتئاب أو الملل **Bored or depressed**
☐ أسباب أخري .... **Other**

Do you sometimes forget taking your medication/insulin? [4]

٢٧. هل تنسي تناول الدواء أو الانسولين؟ [4] ☐ نعم **Yes** ☐ لا **No**

Do you take the right insulin dose? [1]

٢٨. هل تأخذ الجرعة الصحيحة [1] للانسولين؟

☐ دائما **Always** ☐ غالبا **Usually** ☐ ٥٠ % من الوقت **50% of time** ☐ أحيانا **Sometimes** ☐ لا **No**

Do you adjust your insulin dose based on your blood sugar measurements? [1]

٢٩. هل تعدل جرعة الانسولين [1] بناء علي قياسات السكر؟

☐ دائما **Always** ☐ غالبا **Usually** ☐ ٥٠ % من الوقت **50% of time** ☐ أحيانا **Sometimes** ☐ لا **No**

Do you suffer from any medication side effects?

٣٠. هل تعاني من الأثار الجانبية للدواء؟ ☐نعم، مثل ماذا؟ .... **Yes, e.g.:** ☐ لا **No**

Are you aware of your medications' side effects? [5] **Yes** **No**
٣١. هل أنت علي معرفة بالأعراض الجانبية لدواء السكر الذي تتناوله؟ [5] ☐ نعم ☐ لا

Do you stop your medication/insulin when not feeling well? [4]
٣٢. هل تتوقف عن تناول الدواء أو الانسولين عندما تشعر بسوء؟ [4] ☐ نعم ☐ لا

Do you stop your medication/insulin when feeling better? [4]
٣٣. هل تتوقف عن تناول الدواء أو الانسولين عندما تشعر بتحسن؟ [4] ☐ نعم ☐ لا

Are you aware of diabetes complications? [5]
٣٤. هل أنت علي علم بالأضرار الناتجة عن إهمال مرض السكر؟ [5] ☐ نعم ☐ لا

Are you aware of diabetes follow up tests and their times? [5]
٣٥. هل تعلم ما هي التحاليل المطلوبة لمتابعة مرض السكر و مواعيدها؟ [5] ☐ نعم ☐ لا

Do you take these tests regularly? [1]
٣٦. هل أنت منتظم في عمل تلك التحاليل؟ [1] ☐ دائما ☐ غالبا ☐ ٥٠ % من الوقت ☐ أحيانا ☐ لا
Always **Usually** **50% of time** **Sometimes** **No**

Have you taken any of the following tests?
٣٧. هل قمت بعمل تلك التحاليل و الفحوصات؟ ☐ وظائف كلي ☐ تحليل بول ☐ فحص قاع عين ☐ دهون في الدم
kidney function **urine test** **eye exam** **Lipid profile**

When were your last tests and how were the results?
٣٨. متي و كيف كانت نتيجة أخر فحص؟ ....

Do you suffer from teeth problems? **Date of last dental visit:**
٣٩. هل تعاني من مشاكل في الأسنان؟........................... تاريخ أخر زيارة لطبيب الأسنان:..............................

Do you check your feet?
٤٠. هل تقوم بفحص قدميك؟ ☐ نعم بنفسي ☐ نعم عند الطبيب ☐لا
Yes, myself **Yes, at doctor's** **No**

How often do you check your feet?
٤١. كم مرة تقوم بفحص قدميك؟ ☐يوميا: ...... مرات ☐ اسبوعيا: ...... مرات ☐شهريا: ...... مرات ☐عند اللزوم
Daily **weekly** **monthly** **upon need**

Are you aware of the type of diabetes you have? [5]
٤٢. ما نوع السكر الذي تعاني منه؟ [5] ☐ النوع الاول ☐ النوع الثاني ☐ غير متأكد
type 1 **type 2** **Not sure**

How do you think diabetes (will) affect(s) your daily life?
٤٣. كيف يؤثر (أو تعتقد أن يؤثر) مرض السكر علي حياتك اليومية؟ ....

What difficulties do you (think you will) experience monitoring your diabetes?
٤٤. ما هي الصعوبات التي تجدها (أو تعتقد أنك ستجدها) لمتابعة مرض السكر؟ ....

Did you suffer a diabetic coma in the last 3 months? **Yes** **No**
٤٥. هل سبق أن أصبت بغيبوبة سكر في الثلاث أشهر الماضية ؟ ☐نعم ☐لا

Do you suffer any eye, foot, kidney or heart complications?
٤٦. هل تعاني من مضاعفات لمرض السكر مثل مشاكل في القدم أو العين أو الكلي أو القلب؟ ....

Does someone at home help you take care of your diabetes?
٤٧. هل يساعدك من في البيت في العناية بمرض السكر؟ ....

Have you received instructions on how to take care of your diabetes? When and where?
٤٨. هل تلقيت تعليمات في السابق عن مرض السكر أو العناية به؟ متي و أين؟ ....

How do you rate your diabetes awareness level? [5]
٤٩. ما مدي معلوماتك عن السكر و العناية به؟ [5] ☐ واعي ☐ مقبول ☐ ضعيف
aware **fair** **Weak**

How do you feel about having diabetes?
٥٠. كيف تشعر و أنت مريض سكر؟ ....

What do you wish to learn about diabetes?
٥١. ما الذي تحب أن تعرفه
عن مرض السكر؟ ☐النظام الغذائي ☐ الرياضة ☐ الأدوية و مضاعافتها
☐ قياس السكر ☐معلومات أخري ....
healthy diet **physical activity** **Medications & side effects**
blood glucose measurement **Other info**

Are you satisfied with your control of your diabetes and blood sugar levels?
٥٢. هل أنت راض عن تحكمك في السكر و نسبه؟ ....

In the last 3 months, did you suffer extreme increase or decrease in your blood sugar levels?
٥٣. هل عانيت من الارتفاع أو الانخفاض الشديد لنسب السكر
في الثلاث أشهر الماضية؟ ☐يوميا: ...... مرات ☐ اسبوعيا: ...... مرات ☐شهريا: ...... مرات ☐غير متأكد ☐لا
Daily **weekly** **monthly** **not sure** **No**

What did you do at times of increase or decrease of your blood sugar levels? [5]
٥٤. ماذا تفعل في حالة الارتفاع أو الانخفاض؟ [5]..

What is your biggest concern about monitoring your diabetes?
٥٥. ما هو أكثر ما يقلقك من السكر و متابعته؟ ....

الغذاء:(Diet)

Are you aware of the healthy diet for diabetics? [5]
٥٦. هل تعلم ما هو النظام الغذائي الصحي لمرضي السكر؟ [5] ☐ نعم ☐ لا
Yes **No**

Do you follow it? [1]
٥٧. هل تتبعه؟ [1] ☐ دائما ☐ غالبا ☐ ٥٠ % من الوقت ☐ أحيانا ☐لا
Always **Usually** **50% of time** **Sometimes** **No**

Are you comfortable with your body weight? **Yes** **No**
٥٨. هل أنت مرتاح بوزنك الحالي؟ ☐نعم ☐لا

Did your weight change in the last year? **Yes** **No**
٥٩. هل تغير وزنك في السنة الماضية؟ ☐نعم ☐لا

How many meals do you have per day?
٦٠. كم وجبة تتناول في اليوم؟ ....

What types of meals and at what times do you have them?
٦١. ما هي نوعية الوجبات و مواعيدها؟ ....

Do you commit to the preset meal times? [1]
٦٢. هل تلتزم بالمواعيد المحددة [1]
للوجبات؟ ☐دائما ☐غالبا ☐ ٥٠ % من الوقت ☐ أحيانا ☐لا
Always **Usually** **50% of time** **Sometimes** **No**

Do you regularly eat snacks between main meals? [1]
٦٣. هل تتناول شئ ما بين الوجبات الأساسية؟ ....
بمواعيد منتظمة؟ [1] ☐ دائما ☐ غالبا ☐ ٥٠ % من الوقت ☐ أحيانا ☐لا
Always **Usually** **50% of time** **Sometimes** **No**

Do you carry something sweet as you leave home? [1]
٦٤. هل تأخذ معك شئ من السكريات [1]
عند الخروج من البيت؟ ☐دائما ☐غالبا ☐ ٥٠ % من الوقت ☐ أحيانا ☐لا
Always **Usually** **50% of time** **Sometimes** **No**

Do you eat out?
٦٥. هل تأكل خارج البيت؟ ☐يوميا: ...... مرات ☐ اسبوعيا: ...... مرات ☐ شهريا: ...... مرات ☐لا
Daily **weekly** **monthly** **No**

Do you drink canned juices or sodas?
٦٦. هل تشرب المشروبات الغازية و العصائر؟ ☐يوميا: ...... مرات ☐اسبوعيا: ...... مرات ☐ شهريا: ...... مرات ☐لا
Daily weekly monthly No

الرياضة / الحركة:(Exercise)

Do you regularly exercise or exert any physical activity? [1, 6]
٦٧. هل تمارس الرياضة [1, 6] أو الحركة بانتظام؟ ☐يوميا: ...... مرات ☐ اسبوعيا: ...... مرات ☐شهريا: ...... مرات ☐عند اللزوم ☐لا
Daily weekly monthly upon need No

Why not?
٦٨. لم لا؟
☐لا تشعر بتحسن ☐ مشغول أو خارج البيت ☐ لا يوجد تشجيع من العائلة
☐ عندما أشعر بالاكتئاب أو الملل ☐ أشعر بالألم و عدم الارتياح
☐ أسباب أخري ....
I don't feel better busy/away from home No support from family
bored or depressed Pain or discomfort
Other

What kind of exercise do you do? Do you walk 30mins a day? [6]
٦٩. ما نوع الرياضة التي تمارسها؟ هل تمارس المشي ٣٠ دقيقة يوميا؟ [6] ....

Do you exercise strenuously? [1]
٧٠. هل تمارس الرياضة [1] أو الحركة بكثافة؟ ☐ دائما ☐ غالبا ☐ ٥٠ % من الوقت ☐ أحيانا ☐ لا
Always Usually 50% of time Sometimes No

Does your health condition affect your ability to be physically active?
٧١. هل تؤثر حالتك الصحية علي قدرتك علي الحركة؟ ....

Are you aware of exercise benefits for diabetics? [5]
٧٢. هل تعلم فوائد الرياضة لمرضي السكر؟ [5] ☐ نعم ☐ لا
yes No

معلومات اجتماعية:(Social information)

Can you afford the costs of treatment?
٧٣. هل تستطيع التكفل بمستلزمات العلاج من أدوية، غذاء، الخ؟ ☐ نعم ☐ لا
yes No

الثقة في القيام بالأشياء:(Confidence in doing things)

How confident are you to: :ما مدي ثقتك في

Not at all slightly moderately highly fully

Eat every 4-5 hrs? [7]
٧٤. أن تأكل الوجبات كل [7]
٤-٥ ساعات؟ كاملة ☐ عالية ☐ متوسطة ☐ قليلة ☐ منعدمة ☐

Commit to your meal plan even when eating with non-diabetics? [7]
٧٥. أن تلتزم بالنظام الغذائى عندما تأكل مع [7]
اخرين غير مصابين بالسكر؟ كاملة ☐ عالية ☐ متوسطة ☐ قليلة ☐ منعدمة ☐

Pick the right type of food when feeling hungry (e.g. between meals)? [7]
٧٦. اختيارك لنوع الأكل المناسب عندما [7]
تشعر بالجوع (ما بين الوجبات مثلا)؟ كاملة ☐ عالية ☐ متوسطة ☐ قليلة ☐ منعدمة ☐

Exercise 30 mins/day? [7]
٧٧. قدرتك علي الرياضة أو الحركة [7]
٣٠ دقيقة يوميا؟ كاملة ☐ عالية ☐ متوسطة ☐ قليلة ☐ منعدمة ☐

Do what's right to keep your blood sugar level from dropping during exercise? [7]
٧٨. قدرتك علي فعل شئ حتي لا [7]
ينخفض سكرك مع المجهود؟ كاملة ☐ عالية ☐ متوسطة ☐ قليلة ☐ منعدمة ☐

Know what you need to do when your blood sugar level is higher or lower than normal? [7]
٧٩. معرفتك لما عليك فعله عند ارتفاع أو [7]
انخفاض سكرك عن المعدل الطبيعي؟ كاملة ☐ عالية ☐ متوسطة ☐ قليلة ☐ منعدمة ☐

Know when you must see your doctor? [7]
٨٠. معرفة متي يتوجب عليك زيارة [7]
الطبيب؟ كاملة ☐ عالية ☐ متوسطة ☐ قليلة ☐ منعدمة ☐

Control your diabetes so that it doesn't stop you from doing anything you like? [7]
٨١. قدرتك في التحكم في السكر حتي [7]
لا يمنعك من القيام بما تحب أن تفعل؟ كاملة ☐ عالية ☐ متوسطة ☐ قليلة ☐ منعدمة ☐

Control your diabetes and avoid its complications?
٨٢. قدرتك في التحكم في السكر حتي
لا تصاب بمضاعفاته؟ كاملة ☐ عالية ☐ متوسطة ☐ قليلة ☐ منعدمة ☐

Let your doctor monitor your diabetes? [8]
٨٣. متابعة الطبيب لسكرك؟ [8] كاملة ☐ عالية ☐ متوسطة ☐ قليلة ☐ منعدمة ☐

How do you rate your level of satisfaction with your doctor monitoring your diabetes? [9]
٨٤. ما مدي رضائك عن متابعة [9]
الطبيب لسكرك؟ كامل ☐ عالي ☐ متوسط ☐ قليل ☐ منعدم ☐

None slight moderate high full

How do you rate your level of satisfaction with your doctor's communication with you? [9]
٨٥. ما مدي رضائك عن تواصل [9]
الطبيب معك؟ كامل ☐ عالي ☐ متوسط ☐ قليل ☐ منعدم ☐

None slight moderate high full

If you are to receive the SMS messages, when would you prefer to get them?

٨٦. ان كنت ضمن المستلمين للرسائل،

في أي وقت تفضل أن تستلمها؟ ☐ صباحا ☐ ظهرا ☐ مساءا

Evening noon morning

Date التاريخ: ....

Signature التوقيع: ....

Superscripted questions are to rate the following:

1 treatment adherence (using the diabetes self-care inventory)
2 rate of hospital/ER visits and stays
3 average frequency of blood glucose measurement
4 medication adherence (using Morisky scale)
5 diabetes knowledge
6 exercise
7 patient confidence (using diabetes empowerment scale short form and Stanford self-efficacy for diabetes)
8 patient confidence in healthcare provider
9 patient satisfaction

برنامج التوعية و المتابعة لمرضي السكر

Diabetes Awareness Program
Post-study Questionnaire

معلومات عامة:(General Information)

Name / **Participant Number**

١. الاسم: .... ٢. رقم المشترك: ....

Body weight and random blood glucose level

٣. الوزن و السكر العشوائي: .... و ....

***************: في الثلاث أشهر الماضية (منذ بدء البرنامج) ***********

In the last 3 months (since start of program):

Did you stop or reduce smoking? — **Yes, I stopped** — **Yes, I reduced by:** — **No**

٤. للمدخنين: هل توقفت عن أو خفضت معدل التدخين؟ ☐ نعم توقفت ☐ نعم خفضت، بمعدل: .... ☐لا

التاريخ الطبى:(Medical History)

Were you diagnosed with other chronic disorders?

٥. هل شخصت بأمراض مزمنة أخري؟ ....

تاريخ السكر:(Diabetes History)

Did you switch from pills to insulin?

٦. هل حولت من حبوب لانسولين؟ ☐ نعم **yes** ☐ لا **No**

How often did you see your diabetes doctor?

٧. كم مرة قمت بزيارة الطبيب بسبب السكر؟ ☐ يوميا: ...... مرات **Daily** ☐ اسبوعيا: ...... مرات **weekly** ☐ شهريا: ...... مرات **monthly** ☐ عند اللزوم **upon need**

Did you commit to seeing your doctor at preset times? [1]

٨. هل التزمت بزيارة الطبيب [1] في المواعيد المحددة؟ ☐ دائما **Always** ☐ غالبا **Usually** ☐ ٥٠ % من الوقت **50% of time** ☐ أحيانا **Sometimes** ☐ لا **No**

How many times were you admitted to the ER or hospital because of your diabetes? [2]

٩. كم مرة قمت بزيارة غرفة الطوارئ (أو تم احتجازك بالمستشفي) بسبب السكر؟ [2] .... مرات

How often did you check your blood sugar? [1, 3]
١٠. كم مرة قمت بقياس [1, 3]
السكر؟ ☐ يوميا: ...... مرات ☐ اسبوعيا: ...... مرات ☐ شهريا: ...... مرات ☐ عند اللزوم ☐ لم أقيس
daily weekly monthly upon need Did not check

Where did you check your blood sugar?
١١. أين قمت بقياس السكر؟ ☐ بالصيدلية ☐ بالمعمل ☐ يوجد جهاز قياس بالبيت
Pharmacy Lab Glucometer at home

Did you record your results? [1]
١٢. هل قمت بتسجيل نتائج القياس؟ [1] ☐ دائما ☐ غالبا ☐ ٥٠ % من الوقت ☐ أحيانا ☐ لا
Always Usually 50% of time Sometimes No

Why didn't you check regularly?
١٣. لماذا لم تقيس السكر باستمرار؟ ☐ لا يوجد تشجيع من العائلة ☐ أكره شك نفسي ☐ سعر التحليل
☐ مشغول أو خارج البيت ☐ لا تشعر بتحسن ☐ عندما أشعر بالاكتئاب أو الملل
☐ أسباب أخري ....
No support from family I hate pricking myself Test cost
busy/away from home I don't feel better Bored or depressed
Other

Usual test result:
١٤. النتيجة المعتادة لتحليل السكر: صائم (قبل الأكل) .... فاطر (بعد الأكل بساعتين) .... عشوائي ....
fasting 2-hrs postprandial Random

Did you take your medications/insulin on time? [1]
١٥. هل انتظمت في مواعيد [1]
الدواء أو الانسولين؟ ☐ دائما ☐ غالبا ☐ ٥٠ % من الوقت ☐ أحيانا ☐ لا
Always Usually 50% of time Sometimes No

Excluding times you forgot, were you sometimes careless taking your medicine/insulin? [4]
١٦. عدا النسيان، هل أهملت أحيانا في تناول الدواء أو الانسولين؟ [4] ☐ نعم ☐ لا
Yes No

At what rate did you miss taking your medications on time?
١٧. بأي معدل كنت غير منتظم
في تناول الدواء في مواعيده؟ ☐ يوميا: ...... مرات ☐ اسبوعيا: ...... مرات ☐ شهريا: ...... مرات
daily weekly Monthly

What made you miss taking your medications on time?
١٨. ما الذي جعلك ☐ أعراض جانبية ☐ لاتشعر بتحسن ☐ مشغول أو خارج البيت
غير منتظم؟ ☐ لا يوجد تشجيع من العائلة ☐ سعر الدواء ☐ عندما أشعر بالاكتئاب أو الملل

☐ أسباب أخري ....

Busy/away from home   I don’t feel better   side effects
Bored or depressed   price of medication   no support from family
Other

Did you sometimes forget taking your medication/insulin? [4]

١٩. هل نسيت أحيانا تناول الدواء أو الانسولين؟ [4]   ☐ نعم   ☐ لا

Yes   No

Did you take the right insulin dose? [1]

٢٠. هل أخذت الجرعة الصحيحة [1] للانسولين؟   ☐ دائما   ☐ غالبا   ☐ ٥٠ % من الوقت   ☐ أحيانا   ☐ لا

Always   Usually   50% of time   Sometimes   No

Did you adjust your insulin dose based on your blood sugar measurements? [1]

٢١. هل عدلت جرعة الانسولين [1] بناء علي قياسات السكر؟   ☐ دائما   ☐ غالبا   ☐ ٥٠ % من الوقت   ☐ أحيانا   ☐ لا

Always   Usually   50% of time   Sometimes   No

Did you become aware of the side effects of your medications? [5]   **Yes   No**

٢٢. هل تعرفت علي الأعراض الجانبية لدواء السكر الذي تتناوله؟ [5]   ☐ نعم   ☐ لا

Did you stop your medication/insulin when you were not feeling well? [4]

٢٣. هل توقفت عن تناول الدواء أو الانسولين عند الشعور بسوء؟ [4]   ☐ نعم   ☐ لا

Did you stop your medication/insulin when you felt better? [4]

٢٤. هل توقفت عن تناول الدواء أو الانسولين عند الشعور بتحسن؟ [4]   ☐نعم   ☐ لا

Did you become more aware of diabetes complications? [5]

٢٥. هل أصبحت أكثر علما بالأضرار الناتجة عن إهمال مرض السكر؟ [5]   ☐ نعم   ☐ لا

Did you become more aware of diabetes follow up tests and their times? [5]

٢٦. هل أصبحت أكثر علما بالتحاليل المطلوبة لمتابعة مرض السكر و مواعيدها؟ [5]   ☐ نعم   ☐ لا

Did you take these tests regularly? [1]

٢٧. هل إنتظمت في عمل تلك التحاليل؟ [1]   ☐ دائما   ☐ غالبا   ☐ ٥٠ % من الوقت   ☐ أحيانا   ☐ لا

Always   Usually   50% of time   Sometimes   No

Did you take any of the following tests?

٢٨. هل قمت بعمل تلك التحاليل و الفحوصات؟   ☐وظائف كلي   ☐ تحليل بول   ☐ فحص قاع عين   ☐ دهون في الدم

kidney function   urine test   eye exam   Lipid profile

When were your last tests and how were the results?

٢٩. متي و كيف كانت نتيجة أخر فحص؟ ....

Did you see your dentist? **Yes** **No**

٣٠. هل قمت بزيارة طبيب الأسنان؟ ☐ نعم .... ☐ لا

Did you check your feet?

٣١. هل قمت بفحص قدميك؟☐ نعم بنفسي ☐ نعم عند الطبيب ☐لا

Yes, myself **Yes, at doctor's** **No**

How often did you check your feet?

٣٢. كم مرة قمت بفحص قدميك؟ ☐يوميا: ...... مرات ☐ اسبوعيا: ...... مرات ☐ شهريا: ...... مرات ☐عند اللزوم

Daily **weekly** **monthly** **upon need**

Did you become aware of the type of diabetes you have? [5]

٣٣. هل أصبحت علي علم بنوع السكر الذي تعاني منه؟ [5] ☐ النوع الاول ☐ النوع الثاني ☐ غير متأكد

type 1 **type 2** **Not sure**

You told us about the effect of diabetes on your daily life. Did it get better?

٣٤. أخبرتنا بتأثير السكر علي حياتك اليومية.
هل من تحسن؟ ☐نعم ☐ بعض الشيء ☐ قليل ☐ غير متأكد ☐لا ☐لم توجد مشكلة

yes **somewhat** **slightly** **not sure** **No** **Had no problem**

You told us about difficulties you experienced monitoring your diabetes? Did they get better?

٣٥. أخبرتنا ببعض الصعوبات لمتابعة السكر.
هل من تحسن؟ ☐نعم ☐ بعض الشيء ☐ قليل ☐ غير متأكد ☐لا ☐لم توجد مشكلة

yes **somewhat** **slightly** **not sure** **No** **Had no problem**

Did you suffer a diabetic coma?

٣٦. هل أصبت بغيبوبة سكر؟ ☐ نعم ☐ لا

Yes **No**

You mentioned suffering some eye, foot, kidney or heart complications. Did they get better?

٣٧. أخبرتنا ببعض المضاعفات في العين، القدم، الكلي أو القلب.
هل من تحسن؟ ☐نعم ☐ بعض الشيء ☐ قليل ☐ غير متأكد ☐لا ☐لم توجد مشكلة

yes **somewhat** **slightly** **not sure** **No** **Had no problem**

Did someone at home help you take care of your diabetes?

٣٨. هل ساعدك من في البيت في العناية بمرض السكر؟ ....

How do you rate your diabetes awareness level now? [5]

٣٩. كيف أصبح مستوي معلوماتك عن السكر؟ [5] ☐واعي ☐ مقبول ☐ ضعيف

aware **fair** **Weak**

Did your knowledge about diabetes and how to care for it improve?
٤٠. هل شعرت بتحسن معلوماتك عن السكر و العناية به؟

نعم ☐ بعض الشيء ☐ قليل ☐ غير متأكد ☐ لا ☐ لم أقرأ ☐

yes somewhat slightly not sure No Did not read SMS or booklet

You told us about the way you felt about having diabetes. Did it get better?
٤١. أخبرتنا بشعورك و أنت مريض سكر. هل من تحسن؟

نعم☐ بعض الشيء☐ قليل☐ غير متأكد☐ لا☐ لم توجد مشكلة☐

yes somewhat slightly not sure No Had no problem

You told us about some things you wished to learn about diabetes. Were we able to give you such information?
٤٢. أخبرتنا بما تحب أن تعرفه عن السكر. هل نجحنا في توصيل المعلومات اليك؟

نعم☐ بعض الشيء ☐ قليل ☐ غير متأكد ☐ لا☐ لم أقرأ ☐

yes somewhat slightly not sure No Did not read SMS or booklet

Were you satisfied with your control of your diabetes and your blood sugar levels?
٤٣. هل كنت راض عن تحكمك في السكر و نسبه؟ ....

Did you suffer extreme increase or decrease in your blood sugar levels?
٤٤. هل عانيت من الارتفاع أو الانخفاض الشديد لنسب السكر؟

☐يوميا: ...... مرات ☐ اسبوعيا: ...... مرات ☐شهريا: ...... مرات ☐غير متأكد ☐لا

Daily weekly monthly not sure No

What did you do at times of increase or decrease of your blood sugar levels? [5]
٤٥. ماذا كنت تفعل في حالة الارتفاع أو الانخفاض؟ [5] ....

You told us about your biggest worries monitoring your diabetes. Did it get better?
٤٦. أخبرتنا بأكثر ما يقلقك من السكر و متابعته. هل من تحسن؟

نعم☐ بعض الشيء☐ قليل☐ غير متأكد☐ لا☐ لم توجد مشكلة☐

yes somewhat slightly not sure No Had no problem

<u>الغذاء:(Diet)</u>

Did you become aware of the healthy diet for diabetics? [5]
٤٧. هل تعرفت علي النظام الغذائي الصحي لمرضي السكر؟ [5] ☐ نعم ☐ لا

Yes No

Did you follow it? [1]
٤٨. هل اتبعته؟ [1] ☐ دائما ☐ غالبا ☐ ٥٠ % من الوقت ☐ أحيانا ☐لا

Always Usually 50% of time Sometimes No

Did your body weight change?

٤٩. هل تغير وزنك؟ ☐ نعم أكثر راحة ☐ نعم أقل راحة ☐ لا

Yes, more comfortable **Yes, less comfortable** **No**

How many meals did you have per day?

٥٠. كم وجبة تناولت في اليوم؟ ....

What types of meals and at what times did you have them?

٥١. ما هي نوعية الوجبات و مواعيدها؟ ☐خطة غذاء ☐ صحي ☐كميات كبيرة ☐ مواعيد ثابتة

☐كل (أي) حاجة ☐ غير صحي ☐كميات معتدلة ☐ مواعيد متغيرة

meal plan **healthy** **big portions** **regular times**

every (any) thing **Unhealthy** **moderate portions** **irregular times**

Did you commit to the preset meal times? [1]

٥٢. هل التزمت بالمواعيد المحددة [1] للوجبات؟ ☐دائما ☐غالبا ☐ ٥٠ % من الوقت ☐ أحيانا ☐لا

Always **Usually** **50% of time** **Sometimes** **No**

Did you regularly eat snacks between main meals? [1]

٥٣. هل تناولت شئ ما بين الوجبات الأساسية؟ .... بمواعيد منتظمة؟ [1] ☐ دائما ☐ غالبا ☐ ٥٠ % من الوقت ☐ أحيانا ☐لا

Always **Usually** **50% of time** **Sometimes** **No**

Did you carry something sweet as you left home? [1]

٥٤. هل أخذت معك شئ من السكريات [1] عند الخروج من البيت؟ ☐دائما ☐غالبا ☐ ٥٠ % من الوقت ☐ أحيانا ☐لا

Always **Usually** **50% of time** **Sometimes** **No**

Did you eat out?

٥٥. هل أكلت خارج البيت؟ ☐ يوميا: ...... مرات ☐ اسبوعيا: ...... مرات ☐ شهريا: ...... مرات ☐لا

Daily **weekly** **monthly** **No**

Did you drink canned juices or sodas?

٥٦. هل شربت المشروبات الغازية أو العصائر؟ ☐ يوميا: ...... مرات ☐ اسبوعيا: ...... مرات ☐شهريا: ...... مرات ☐لا

Daily **weekly** **monthly** **No**

<u>الرياضة / الحركة:(Exercise)</u>

Did you regularly exercise or exert any physical activity? [1, 6]

٥٧. هل مارست الرياضة [1, 6] أو الحركة بانتظام؟ ☐يوميا: ...... مرات ☐ اسبوعيا: ...... مرات ☐شهريا: ...... مرات ☐عند اللزوم ☐لا

No upon need monthly weekly Daily

Why not?
٥٨. لم لا؟ ☐ لا تشعر بتحسن ☐ مشغول أو خارج البيت ☐ لا يوجد تشجيع من العائلة
☐ عندما أشعر بالاكتئاب أو الملل ☐ أشعر بالألم و عدم الارتياح
☐ أسباب أخري ....
I don't feel better busy/away from home No support from family
bored or depressed Pain or discomfort
Other

What kind of exercise did you do? Did you walk 30mins a day as we instructed? [6]
٥٩. ما نوع الرياضة التي مارستها؟ هل مارست المشي ٣٠ دقيقة يوميا كما نصحناك؟ [6] ....

Did you exercise strenuously? [1]
٦٠. هل مارست الرياضة [1]
أو الحركة بكثافة؟ ☐ دائما ☐ غالبا ☐ ٥٠ % من الوقت ☐ أحيانا ☐ لا
Always Usually 50% of time Sometimes No

Did you become aware of exercise benefits for diabetics? [5]
٦١. هل تعرفت علي فوائد الرياضة لمرضي السكر؟ [5] ☐ نعم ☐ لا
yes No

<u>الثقة في القيام بالأشياء:(Confidence in doing things)</u>

كيف أصبحت ثقتك في::How confident were you to:

	fully	highly	moderately	slightly	Not at all
Eat every 4-5 hrs? [7]<br>٦٢. أن تأكل الوجبات كل [7] ٤-٥ ساعات؟	☐كاملة	☐عالية	☐متوسطة	☐قليلة	☐ منعدمة
Commit to your meal plan even when eating with non-diabetics? [7]<br>٦٣. أن تلتزم بالنظام الغذائى عندما تأكل مع [7] اخرين غير مصابين بالسكر؟	☐كاملة	☐عالية	☐متوسطة	☐قليلة	☐منعدمة
Pick the right type of food when feeling hungry (e.g. between meals)? [7]<br>٦٤. اختيارك لنوع الأكل المناسب عندما [7] تشعر بالجوع (ما بين الوجبات مثلا)؟	☐كاملة	☐عالية	☐متوسطة	☐قليلة	☐منعدمة
Exercise 30 mins/day? [7]<br>٦٥. قدرتك علي الرياضة أو الحركة [7] ٣٠ دقيقة يوميا؟	☐ كاملة	☐عالية	☐ متوسطة	☐قليلة	☐ منعدمة
Do what's right to keep your blood sugar level from dropping during exercise? [7]<br>٦٦. قدرتك علي فعل شئ حتي لا [7] ينخفض سكرك مع المجهود؟	☐كاملة	☐عالية	☐متوسطة	☐قليلة	☐منعدمة

Know what you need to do when your blood sugar level is higher or lower than normal? [7]

٦٧. معرفتك لما عليك فعله عند ارتفاع أو [7] انخفاض سكرك عن المعدل الطبيعي؟ ☐ كاملة ☐ عالية ☐ متوسطة ☐ قليلة ☐ منعدمة

Know when you must see your doctor? [7]

٦٨. معرفة متي يتوجب عليك زيارة [7] الطبيب؟ ☐كاملة ☐عالية ☐متوسطة ☐قليلة ☐منعدمة

Control your diabetes so that it doesn't stop you from doing anything you like? [7]

٦٩. قدرتك في التحكم في السكر حتي [7] لا يمنعك من القيام بما تحب أن تفعل؟ ☐ كاملة ☐ عالية ☐ متوسطة ☐قليلة ☐ منعدمة

Control your diabetes and avoid its complications?

٧٠. قدرتك في التحكم في السكر حتي لا تصاب بمضاعفاته؟ ☐ كاملة ☐ عالية ☐ متوسطة ☐ قليلة ☐ منعدمة

Let the hospital monitor your diabetes? [8]

٧١. متابعة المستشفي لسكرك؟ [8] ☐ كاملة ☐ عالية ☐ متوسطة ☐ قليلة ☐منعدمة

How do you rate your level of satisfaction with the hospital monitoring your diabetes? [9]

٧٢. مدي رضائك عن متابعة [9] المستشفي لسكرك؟ ☐ كامل ☐ عالي ☐ متوسط ☐ قليل ☐ منعدم

full high moderate slight None

How do you rate your level of satisfaction with our communication with you? [9]

٧٣. مدي رضائك عن تواصلنا [9] معك؟ ☐ كامل ☐عالي ☐ متوسط ☐قليل ☐منعدم

full high moderate slight None

<u>(Your opinion of the program):رأيك في البرنامج</u>

How did you feel being part of the program?

٧٤. كيف كان شعورك و أنت عضو في البرنامج؟ ....

Did you get the messages every day? Yes No, why not?

٧٥. هل وصلتك الرسائل يوميا؟ ☐ نعم ☐ لا، لم لا؟ ....

Were the SMS receipt times convenient?

٧٦. هل كان وقت استلام الرسائل مناسبا ؟ ☐ نعم ☐ لا، لم لا؟ ....

Was the content clear and understandable?

٧٧. هل كان المحتوي واضح و مفهوم؟ ☐ نعم ☐بعض الشيء ☐ لا، لم لا؟ ....

Somewhat

Did you experience any difficulty reading and understanding the messages?

٧٨. هل واجهتك صعوبة في قراءة و فهم الرسائل؟ ☐ نعم .... ☐ لا

Yes, please explain No

How did you find the daily SMS sending frequency?
٧٩. كيف وجدت المعدل اليومي لاستلام الرسائل؟ ....

Did you think there was anything missing in the content of the messages?
٨٠. في رأيك هل من شئ كانت تنقصه الرسائل؟ ....

Did you read the information booklet? **Yes** **No, why not?**
٨١. هل قرأت كتيب المعلومات؟ ☐ نعم ☐ لا، لم لا؟ ....

How was it? Was it clear and understandable?
٨٢. كيف وجدته؟ هل كان محتواه واضح و مفهوم؟ ....

Would you prefer the booklet or the SMSs? Why? **SMSs** **Booklet**
٨٣. هل تفضل الرسائل أم الكتيب و ما السبب؟ ☐ الرسائل .... ☐ الكتيب ....

Are you generally satisfied with the program? [9] **Yes** **No, why not?**
٨٤. بشكل عام هل أنت راض عن البرنامج و خدماته؟ [9] ☐ نعم ☐ لا، لم لا؟ ....

If it continues, would you stay enrolled with us? [9]
٨٥. هل ستستمر معنا إن إستمر البرنامج؟ [9] ☐ نعم ☐ لا، لم لا؟ ....

Would you recommend it to others? [9]
٨٦. هل سترجح الإشتراك في البرنامج للآخرين؟ [9] ☐ نعم ☐ لا، لم لا؟ ....

Do you think there's anything missing in the program?
٨٧. في رأيك هل من شئ ينقصه البرنامج؟ ....

Do you think the program could improve the hospital's reputation?
٨٨. هل تظن أن وجود البرنامج يحسن من سمعة المستشفي؟ ☐ نعم ☐ لا، لم لا؟ ....

Signature
التوقيع: ....

Date
التاريخ: ....

Superscripted questions are to rate the following:

1 treatment adherence (using the diabetes self-care inventory)
2 rate of hospital/ER visits and stays
3 average frequency of blood glucose measurement
4 medication adherence (using Morisky scale)
5 diabetes knowledge
6 exercise
7 patient confidence (using diabetes empowerment scale short form and Stanford self-efficacy for diabetes)
8 patient confidence in healthcare provider
9 patient satisfaction
