# Supplementary material for: SMS education for the promotion of diabetes self-management in low & middle income countries: a pilot randomized controlled trial in Egypt
Source: BMC Public Health. 2017 Dec 19;17:962. doi: 10.1186/s12889-017-4973-5 (PMC5735794; doi:10.1186/s12889-017-4973-5)
Supplement: Supplementary file 2 — Instruction booklet and nutrition leaflet; both the instruction booklet and nutrition leaflet as given to patients followed by their English translation. (PDF 860 kb) [file 12889_2017_4973_MOESM2_ESM.pdf]

برنامج التوعية و المتابعة لمرضي السكر DiabAwPro

"كتيب التعليمات"

التعرف على مرض السكر والمشاركة بنشاط في العلاج أمر حيوي لمرضى السكر، حيث أن مضاعفات المرض تكون أقل شيوعا وأقل خطورة لدى الأشخاص المتحكمين في مستويات السكر في الدم بشكل جيد. في هذا الكتيب ستجد معلومات و نصائح عامة لمرضي السكر. قم باستشارة الطبيب ان كانت لديك شكوك عن مدي ملائمتها لك. الهدف هو التدريب علي تقليل نسبة الهيموجلوبين السكري حيث أن التحكم في هذه النسبة قد يمنع أو يقلل من حدوث مضاعفات.

معلومات عامة عن مرض السكر:

- مرض السكر مرض خطير ولكن التحكم فيه بشكل سليم يمنع أو يؤخر ظهور مضاعفات
- يمكن التحكم في مرض السكر بالحفاظ علي نظام غذائي صحي، و الحركة اليومية، و الامتناع عن التدخين، والحفاظ على وزن الجسم الطبيعي، والتحكم في ضغط الدم، والعناية بالقدمين، وتناول الأدوية في الأوقات المحددة
- يجب الحفاظ علي السكر الصائم ما بين ٧٠ و ١٣٠، و العشوائي و بعد الأكل بساعتين أقل من ١٨٠، و الهيموجلوبين السكري حول ٧%. قد تتغير هذه المستويات مع السن و سنين الاصابة بالمرض و إذا كنت تعاني من مضاعفات
- التدخين، الكولسترول، السمنة، ارتفاع ضغط الدم، وعدم ممارسة الرياضة بانتظام لهم تأثير سلبي علي مرض السكر
- خفض نسبة الهيموجلوبين السكري يقلل من فرصة الإصابة بمضاعفات كالسكتة الدماغية و أمراض الكلي و العيون
- تعطيك عينة الهيموجلوبين السكري في المعمل معلومات حول ضبط السكر لديك لمدة شهرين او ثلاثة. عندما تكون اقل من ٧% يعتبر السكر منضبط بشكل جيد جداً. أما عندما تكون ما بين ٧% الى ٧,٥% يعتبر السكر منضبط بشكل جيد. يحدد الهدف لكل شخص على حدة حسب السن و عوامل أخري يحددها الطبيب
- لا تتردد أن تسأل طبيبك إذا كان هناك أي شيء ترغب في تعلمه أو معرفته عن مرض السكر
- السكر ليس معديا و لكنه مرض مزمن. فلا تتوقع الشفاء بعد فترة من العلاج. بل يجب عليك التعايش معه و التحكم فيه

الغذاء:

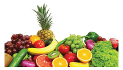

- الغذاء الصحي لمرضى السكر عموما هو نفسه الغذاء الصحي لأي شخص - قليل الدهون، معتدل في الملح والسكر، مع وجبات تحتوي علي البروتين الطري والخضروات غير النشوية والحبوب الكاملة و الفاكهة
- النشويات يمكن أن تكون جزء من خطة الغذاء، ولكن بالكميات المناسبة! يمكن تناول الخبز والمكرونة والأرز والخضروات النشوية مثل البطاطس والذرة في وجبات الطعام والوجبات الخفيفة
- يمكن أن تؤكل الحلويات كجزء من خطة الغذاء الصحية و الي جانب ممارسة التمارين الرياضية. فانها ليست ممنوعة لمرضى السكر أكثر من الأشخاص غير المصابين بالسكر إن كان تناولها بكميات صغيرة و فقط في المناسبات الخاصة حتى يتمكن المريض من التركيز على الأغذية الصحية
- الفاكهة مفيدة للصحة و لكن الاكثار منها قد يضر! اسأل طبيبك عن الأنواع و الكميات الصحية المناسبة لك
- طريقة الأكل الصحية تعني محاولة عدم تناول الكثير من الطعام، عدم الإكثار من نوع واحد من الغذاء، المباعدة بين وجبات الطعام بالتساوي على مدار اليوم، وعدم تخطي أي وجبة
- تناول الطعام على فترات منتظمة (مثلا كل ٤-٥ ساعات)، وإن شعرت بالجوع بين الوجبات، فتناول وجبة خفيفة. إتفق مع طبيبك علي خطة الغذاء الصحية و الوجبات الخفيفة المناسبة لك
- تجنب المشروبات السكرية مثل المشروبات الغازية و العصائر و مشروبات الطاقة والشاي المحلى ... الخ. فانها ترفع من نسبة الجلوكوز في الدم وتضيف عدة مئات من السعرات الحرارية في حصة واحدة فقط!
- الوجبة المثالية تتكون من أحد الأطعمة المحتوية على نشويات مع خضراوات طازجة و لحم منزوع الدسم أو سمك و قليل من المواد الدهنية و احدى منتجات الألبان مع فاكهة و مياه معدنية أو غازية كمشروب
- تتوقف التغذية السليمة على التسوق بشكل صحيح! فلا يجب اعداد طعاما خاصا لمريض السكر، بل الأسرة كلها مدعوة لتأكل بطريقة صحية!
- إذا كنت تأكل خارج البيت فحافظ على القواعد الأساسية للتغذية السليمة واتبع الإرشادات المتفق عليها مع طبيبك!
- من الضروري معرفة كيفية تحديد السكريات و قياس كمياتها في وجبات الطعام. اسأل طبيبك عن كيفية القياس!
- ليس بالضرورة أن يفيدك كل ما هو صحي. فالعسل صحي ولكنه ليس جيدا للسيطرة على مرض السكر. و التمور مفيدة لكنها لا يمكن أن تؤكل بكميات كبيرة. اسأل طبيبك ان كانت لديك شكوك حول ما هو صحي و مفيد

نصائح:

- احمل دائما أحد السكريات التي ترفع السكر سريعا كالعصير أو حبوب الجلوكوز وخاصة عند المشي أو بذل الجهد
- إن كنت تجلس لساعات طويلة في العمل أو المنزل، فحاول النهوض مرة كل ساعة و المشي حول الغرفة
- ان شعرت بتحسن أو سوء فلا تتوقف عن تناول الأدوية. بل تحدث مع طبيبك عن ما تشعر به وما يجب عليك فعله
- اسأل طبيبك عن مضاعفات السكر المحتملة. فالتعلم عن المرض خطوة أساسية للمشاركة بفعالية في العلاج
- إن واجهت مشكلة في العناية بمرض السكر فعليك اشراك طبيبك أو أفراد الأسرة. فذلك قد يوفر لك دعما كبيرا
- اسأل طبيبك عن ما يجب أن تفعله في حالات ارتفاع أو انخفاض السكر في الدم
- مارس الرياضة بانتظام حسب تعليمات الطبيب و إسأله عن التمارين المناسبة لك و فوائد الرياضة لمرضي السكر
- ما لم يخالف ذلك تعليمات الطبيب، فالجدول الجيد لممارسة الرياضة هو ٤-٥ مرات اسبوعيا ١٥-٣٠ دقيقة يوميا
- اسأل طبيبك عن الحالات الاستثنائية التي يجب عليك أن تذهب لرؤيته ان واجهتها
- فلتكن لديك الرغبة في السيطرة على مرض السكر حتي تتجنب مضاعفاته
- إذا أخذت جرعة زائدة من الانسولين فشعرت بهبوط مع عرق بارد فعليك أن تشرب بعض العصير
- اعتمد على نفسك لتعديل حالتك الصحية! فمثلا عليك اتباع طريقة اخرى للتغذية و ممارسة الرياضة يوميا و بالطبع التوقف عن التدخين بالنسبة للمدخنين. فهدفك هو: ادوية اقل، وزيادة في التنظيم الغذائي و التمرينات الرياضية
- الحركة اليومية ضرورية للجميع! فهي تساعد علي فقدان الوزن و تقوية أوعية القلب
- ينصح بالمتابعة الذاتية للسكر بالبيت أو الصيدلية و بعد تحليل المعمل وسيلة لضمان دقة الجهاز و قدرتك علي القياس
- ينصح بالاحتفاظ بعلبة شرائط تحليل البول بالبيت
- ينصح لكل مريض سكر بالاحتفاظ بجرعة انسولين بالبيت للطوارئ

الارتفاع أو الانخفاض في السكر:

- يرتفع السكر بسبب عدم الالتزام بمواعيد الدواء أو الأنسولين أو خطة الغذاء أو الرياضة و تكون نتائج القياس أعلي من ١٣٠ صائم، ١٨٠ بعد الأكل بساعتين أو عشوائي، و ٧% هيموجلوبين سكري مع شعور بالعطش و تبول زائد
- ينخفض السكر بسبب الإفراط في استخدام الأنسولين أو الأدوية، أو ترك وجبة من الوجبات المحددة، أو بذل المجهود البدني الزائد. و تكون نتيجة القياس أقل من ٧٠ مع شعور برعشة و تعرق زائد
- الارتفاع أو الانخفاض الشديد في مستويات السكر في الدم قد يؤدي الي غيبوبة سكر
- الجلوكاجون قد ينقذ حياتك! ينبغي علي كل مريض سكر، خاصة ان كان يعاني من نقص السكر في الدم أو يعتمد في علاجه علي الانسولين، أن يحتفظ بطقم الجلوكاجون بالمنزل
- ينصح بتناول العصير أو الحلوي عند الهبوط المفاجئ للسكر و شرب الكثير من الماء و أخذ جرعة انسولين استثنائية عند الارتفاع المفاجئ. اسأل طبيبك عن هذه الجرعة. ولا تقلق! ان كنت لا تأخذ الانسولين فأخذه استثنائيا لا يعني انك ستأخذه دائما. فهو ليس بادمان. ولكن احترس! ان تقيأت كثيرا مع ارتفاع السكر فاذهب الي المستشفي علي الفور

الرعاية عند الاصابة بأمراض أخرى:

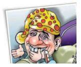

- الاصابة بالبرد أوالانفلونزا يمكن أن ترفع نسبة السكر في الدم
- افحص مستوى السكر في الدم أكثر من المعتاد عند الاصابة بالبرد أو الانفلونزا
- لا يجب أن تتوقف الأدوية أثناء الاصابة بأمراض أخري الا اذا نصحك الطبيب بذلك
- ينبغي اختبار الكيتونات في البول عند الاصابة بأمراض أخري أو في فترات ضعف السيطرة علي السكر

تأثير السكر على حياتك الشخصية و الاجتماعية:

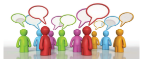

- إذا كان مرض السكر يسبب لك الخوف أو الغضب أو التوتر، فتذكر أن هذا أمر طبيعي. يمكنك التحدث مع طبيبك أو العائلة أو الأصدقاء و ضمهم الي نمط حياتك الصحي ودعوتهم لحضور مواعيد الطبيب معك
- فلتكن لديك العزيمة في الالتزام بخطة الغذاء حتى عند تناول الطعام مع آخرين غير مصابين بالسكر
- فلتكن لديك العزيمة في السيطرة على مرض السكر حتي لا يمنعك من فعل الأشياء التي تحبها
- مرض السكر ليس بعقبة في طريق الحياة الطبيعية أوالزواج

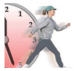

الحركة و الرياضة:

- ممارسة النشاط جزء آخر ذو أهمية للتحكم في السكر. أي نوع من النشاط البدني يساعد على خفض السكر في الدم
- بغض النظر عن نوع مرض السكر الذي تعاني منه، فممارسة النشاط البدني بانتظام أمر لا غني عنه!
- تحدث مع طبيبك إن كان لديك تساؤلات حول الأنشطة المناسبة لك. فمن أنواع النشاط البدني المختلفة المشي، والسباحة، وصعود الدرج بدلا من استخدام المصعد، ..... الخ
- إن كنت غير معتاد ممارسة النشاط، فيمكنك البدء بعشر دقائق من المشي يوميا وإضافة خمس دقائق كل أسبوع. الهدف الجيد هو ٣٠ دقيقة يوميا
- يمكنك أيضا تقسيم النشاط! مثلا المشي ١٠ دقائق قبل أو بعد وجبات الطعام بدلا من ٣٠ دقيقة دفعة واحدة، أو ١٥ دقيقة من التمارين الرياضية في الصباح قبل العمل و ١٥ دقيقة أخرى عندما تعود الي المنزل
- المشي وسيلة رائعة للحصول علي اللياقة! فهو يحسن ضغط الدم والكولسترول والتوتر والاكتئاب. كما يساعد علي فقدان الوزن ويقلل من خطر الإصابة بأمراض القلب. ادعو الأخرين للانضمام لك لجعل المشي أكثر متعة!
- اسأل طبيبك عن ما يجب عليك القيام به حتي لا ينخفض سكرك أثناء المجهود البدني
- لا تمارس الرياضة أو المجهود البدني إن كان السكر منخفضا أو مرتفعا (أعلي من ٢٤٠) ويوجد كيتونات في البول

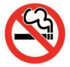

التدخين:

- يجب ألا يقتصر العلاج علي خفض السكر بل يجب أيضا تصحيح عوامل الخطر كالتدخين والسمنة و الضغط العالي
- التدخين من أخطر التهديدات لقدميك! فهو يساعد علي تصلب الشرايين بشكل أسرع و يقلل من تدفق الدم الي القدمين مما يؤخر شفاء الجروح. معظم مرضي السكر الذين يحتاجوا الي بتر جراحي هم من المدخنين
- احذر! احتمال الاصابة بمضاعفات السكر مثل السكتة الدماغية يرتفع إذا كنت تدخن أو إن لم تكن تمارس النشاط

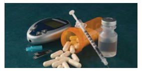

دواء السكر و أثاره الجانبية:

- إن كنت تعاني من مرض السكر من النوع الثاني و قال لك الطبيب أنك تحتاج إلى استخدام الأنسولين، فإن هذا لا يعني أنك فشلت في التحكم في مرض السكر. إنما يعني فقط أن قدرة جسمك علي إنتاج الأنسولين بدأت تقل تدريجيا، و أصبحت الأدوية بمفردها غير كافية للحفاظ على نسبة طبيعية للسكر في الدم. عند إذ يكون استخدام الانسولين للحصول على نسبة صحية للسكر أمر جيد وليس سيئ
- عند البدء في تناول أدوية جديدة، اسأل طبيبك عن الآتي: كم من الأقراص يجب أن تأخذ؟ متي يجب أن تأخذها؟ هل يجب أخذ الدواء على معدة فارغة أم بعد الأكل؟ ماذا يجب أن تفعل إن نسيت أخذ الدواء وتذكرت لاحقا؟ ما هي الآثار الجانبية للدواء؟ ماذا عليك أن تفعل ان عانيت منها؟ هل سيسبب لك الدواء أي مشاكل مع أدوية أخري تتناولها؟
- ان عانيت من الاثار الجانبية للدواء فلا تتوقف عن تناوله و قم باستشارة الطبيب
- الدواء لا يمكن أن يحل محل الرياضة و النظام الغذائي. و لهذا يتوقف علاج السكر عليك أنت أكثر من الأدوية التي يصفها لك الطبيب. سيكون الدواء بلا فائدة إن لم تتبع نظام غذائي مناسب، لأنه سيفقد جزءا كبيرا من فاعليته
- لا تعتمد قوة وفعالية الأدوية علي سعر الدواء
- يحفظ الأنسولين غير المستخدم في الثلاجة ولا يجب أبدا تجميده. و يحفظ الأنسولين المستخدم عند درجة حرارة الغرفة

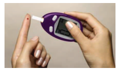

التحاليل و قياس السكر:

- يقاس السكر عادة قبل و بعد الوجبات و عند النوم و قبل بذل المجهود
- يعتبر قياس السكر بالبيت جزء مهم من التحكم في مرض السكر
- القياس يخبرك بمدي تأثير الغذاء والرياضة والأدوية على السكر في الدم و يؤكد لك أنه لا يرتفع أو ينخفض بشدة
- سجل نتائج القياس و اسأل عن مواعيده و نتائجه المستهدفة. سيقرر الطبيب بناءا على خطة العلاج و نوع السكر الذي تعاني منه. فينصح للمتناولين للأنسولين بالقياس ٣ مرات يوميا علي الأقل. أما غير المتناولين فعليهم سؤال الطبيب
- كجزء من الروتين اليومي، تساعد المتابعة الذاتية للسكر علي أختيار نمط الحياة الصحي والعلاج المناسب و اكتشاف أعراض ارتفاع أو انخفاض السكر. كما تساعد طبيبك على تغيير العلاج لمنع حدوث مضاعفات على المدى الطويل
- إن ما تشعر به ليس دائما دليل جيد أو دقيق على ما يحدث. قم بقياس السكر بانتظام وليس فقط عندما تشعربسوء!
- ينصح بشراء جهاز قياس ان كنت تستطيع فهو يساعدك على متابعة السكر والتكيف مع العلاج بناءا على النتائج

- ينصح بعمل تحليل الهيموجلوبين السكري كل ٣ شهور و تحليل بول و قياس وظائف الكلي و فحص قاع العين كل عام حتي لو كنت لا تشكو من شيء
- العلاج لا يجب أن يتوقف قبل قياس السكر أو قبل عمل التحاليل الطبية
- اختبار السكر فى الدم هو أكثر إفادة وأكثر أمانا من اختبار البول

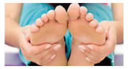

الاهتمام بالقدم:

- يضعف السكر تدفق الدم في القدم والساق! لكن يمكنك تجنب بعض المسببات لذلك كالتدخين وضغط الدم والكولسترول
- يجب ارتداء الأحذية المناسبة و معالجة إصابات الجلد الطفيفة على الفور لتجنب الإصابة بإلتهابات خطيرة
- قد لا تؤلمك القرح لكن يجب أن يفحص الطبيب كل قرحة في الحال لتجنب إلتهابات قد تؤدي إلى فقدان أحد الأطراف
- مرضي السكر أكثر عرضة للبتر الجراحي للقدم من غيرهم. ولكن يمكن الوقاية من ذلك بالمتابعة المستمرة للسكر
- إن أفضل وسيلة لرعاية قدميك تكون بالنظر إليهم وغسلهم كل يوم

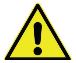

مضاعفات السكر:

- اهمال السكر يعجل من تصلب الشرايين، و قد يؤدي إلى أمراض القلب والسكتات الدماغية و مشاكل في الأقدام
- عدم التحكم في السكر قد يؤدي إلى تلف العين وضعف الرؤية و مشاكل في اللثة و الأسنان
- عدم التحكم في السكر قد يؤدي إلى تلف الكلى الذي قد يتطلب غسيل للكلى في نهاية المطاف
- عدم التحكم في السكر قد يسبب تلف في الأعصاب و يؤدي إلى فقدان الإحساس في القدمين. عند ذلك قد لا يتمكن المريض من ملاحظة إصابات القدم و قد تؤدي إلى التهابات خطيرة تستلزم بتر جراحي للقدم أو الأصابع
- مضاعفات السكر يمكن الوقاية منها أو تأخير الاصابة بها من خلال السيطرة على مستويات السكر في الدم
- التحكم الجيد في الهيموجلوبين السكري يقلل من خطر الاصابة بمشاكل في القدم. و السيطرة المحكمة على السكر تقلل من الاصابة بمضاعفات في الكلى والأعصاب والعين والقلب. السيطرة المحكمة تعني الحفاظ علي السكر الصائم ما بين ٧٠ و ١٢٠ ، ١٦٠ بعد الوجبات و هيموجلوبين سكري أقل من ٧%

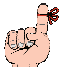

تذكير:

- لا تنسي زيارة الطبيب في المواعيد المحددة و زيارة طبيب الأسنان كل ستة أشهر
- قم بقياس السكر بانتظام بالبيت. ان لم يكن لديك جهاز قياس بالبيت فاذهب الي أقرب صيدلية أو توجه الي عيادة الباطنة بمستشفي الجامعة مرة كل اسبوع. لا تنسي تسجيل نتائج القياس!
- تناول جرعة الدواء أو الانسولين الصحيحة في الأوقات المحددة. لا تهمل في مواعيد الدواء و لا تترك أي جرعة
- ان كنت تأخذ الانسولين فلا تنسي تعديل الجرعة بناء علي نتائج قياس السكر اذا نصحك الطبيب بذلك
- أسأل طبيبك عن الأعراض الجانبية المحتملة للأدوية التي تأخذها وما يجب عليك فعله ان تعرضت لأي منها
- متابعة الوزن هى عنصر لا غنى عنه فى متابعة مستويات السكر. فلا تنسي قياس وزنك مرة فى الأسبوع أو علي الأقل مرة فى الشهر. فيجب عليك معرفة وزنك الحالى و الوزن المثالى لك و أكبر وزن وصلت إليه

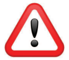

تحذيرات:

- مرضي السكر هم أكثر عرضة للاصابة بسكتة دماغية من غير المصابين بالسكر
- احذر أثناء ممارستك للتمارين الرياضية إن كنت تعاني من أمراض أخرى كارتفاع ضغط الدم أو إذا كنت تعاني من مضاعفات السكر. اسأل طبيبك عن أنواع وفترات التمارين الرياضية المناسبة لك
- تعتبر زيادة الوزن العدو الاول لمرض السكر فحافظ علي نظامك الغذائي و الوزن المثالي الذي حدده لك الطبيب
- عدم الاهتمام بالسكر قد يؤدي الي اصابة الشرايين الصغري مما يؤثر علي العين و الكلي و الأعصاب
- احذر! أخذ الدواء أو الأنسولين في المواعيد المحددة لا يعني أنك يمكن أن تأكل كما تريد
- احذر! السمنة ليست علامة على الصحة الجيدة أوالرخاء الاجتماعي
- احذر! وضع قدميك بالقرب من المدفأة في الطقس البارد لا يحسن من تدفق الدم

Diabetes Awareness Program

"Instruction booklet"

Learning about diabetes and actively participating in the treatment is vital for diabetic patients, since the complications of diabetes are far less common and less severe in people who have well-managed blood sugar levels. In this booklet you will find general information and advice that fit most diabetic patients. Please consult with your doctor if you are in doubt about their suitability for you. Our target is to guide you through lowering your HbA1c levels as glycemic control can prevent or reduce diabetes complications.

Diabetes knowledge:

- Diabetes is a serious disease but managing it properly can prevent or delay its complications.
- Diabetes can be managed by having a healthy diet, regular physical exercise, not smoking, staying at a normal body weight, blood pressure control, proper foot care and taking your medications on time.
- For good diabetes control your fasting/pre-meals sugar levels should be kept in the normal range (between 70 and 130), random/2 hours post-meals under 180, and according to the American Diabetes Association, HbA1c levels around 7 (optimal=6.5). These ranges may change depending on your age, years of diabetes and whether you suffer from diabetes complications.
- Smoking, elevated cholesterol levels, obesity, high blood pressure, and lack of regular exercise can make diabetes worse.
- Lowering your HbA1c levels can reduce your chance of suffering diabetes complications such as stroke, kidney and eye disease.
- The HbA1c test is a lab test that provides you with information on your blood sugar levels in the past 2-3 months. If your HbA1c is less than 7%, this means that you have optimal diabetes control. If it is between 7 and 7.5%, it means that you have good control. The target range is set individually based on your age and other factors determined by your doctor.
- Do not hesitate to ask your doctor if there is anything you would like to learn or know about diabetes.
- Diabetes is not contagious but it is a chronic disease. Cure is not to be expected after a short course of treatment. You have to learn to live with and control it.

Diet:

- A healthy diet for diabetic patients is generally the same as a healthy diet for anyone; low in fat, moderate in salt and sugar, with meals based on lean protein, non-starchy vegetables, whole grains, and fruit.
- Starchy foods can still be part of a healthy meal plan, but in appropriate portions! Whole grain breads, pasta, rice and starchy vegetables like potatoes and corn can be included in your meals and snacks.
- Sweets and desserts can be eaten as part of a healthy meal plan and combined with exercise. They are no more "off limits" to diabetics than they are to people without diabetes. The key to sweets is to have a very small portion and save them for special occasions so that you can focus your meals on healthier foods.

- Fruits are healthy, but this does not mean you can have as much as you wish! Talk to your doctor about the amount, frequency and types of fruits you should eat.
- Healthy eating means trying not to eat too much food; trying not to eat too much of one type of food; spacing your meals evenly throughout the day; and not skipping any meals.
- Eat at regular intervals (e.g., every 4-5 hrs) and if you feel hungry between meals, take a snack. Agree with your doctor on a healthy meal plan and the proper snacks for you.
- Avoid sugary drinks like regular soda, fruit punch, fruit drinks, energy drinks, sweet tea, etc. These will raise your blood glucose and provide several hundred calories in just one serving!
- An ideal meal consists of foods containing starches with fresh vegetables and low-fat meat or fish, a few of fatty substances, and a dairy product with fruit and mineral water or a soft drink.
- Good nutrition depends on shopping for the right things. Special foods must not be prepared for diabetics, but the whole family is invited to eat in a healthy way!
- If you are eating out, keep the ground rules of good nutrition in mind and follow the guidelines you've agreed on with your doctor.
- It is necessary to know how to identify and measure the quantity of sugar in your meals. Ask your doctor about the way to measure.
- Things that are healthy may not be good for you. Honey is healthy but it is not good for diabetes control. Also, dates are healthy but they cannot be eaten in large quantities. Ask your doctor if you’re having doubts about what’s good and healthy for you.

<u>Physical activity:</u>

- Being active is another part of managing your diabetes. Any type of physical activity you do helps lower your blood glucose.
- Regardless of the type of diabetes you have, regular physical activity is essential.
- Talk to your doctor if you have questions about which activities are right for you. Examples of different types of physical activity include walking, swimming, taking the stairs instead of an elevator, etc.
- If you're not used to being active, you can start with 10 minutes of walking each day and add five minutes each week. A good goal is 30 minutes per day.
- You can also split up your activity, e.g. walk 10 minutes before or after meals instead of 30 minutes at once, or 15 minutes of aerobics in the morning before work and another 15 minutes when you get home.
- Walking is a great way to get fit. It improves blood pressure, cholesterol, stress, and depression. It also promotes weight loss and reduces your risk of heart disease. Invite others to join you to make it more pleasant!
- Ask your doctor about what you should do so that your blood sugar won’t drop during physical exertion.
- Do not exercise if your blood sugar is low. Also, do not exercise if your blood sugar is high (> 240) and your urine contains ketones.

<u>Smoking cessation:</u>

- Treatment should not only consider lowering blood glucose levels but should also correct other risk factors such as smoking, obesity and hypertension.

- Smoking is one of the biggest threats to your feet! It makes arteries harden faster and causes decreased blood flow to the feet which makes wounds heal slowly. Many diabetics who need amputations are smokers.
- Careful! Your chances of suffering diabetes complications such as strokes get higher if you smoke or if you are not physically active.

Medications and side effects:

- If you have type 2 diabetes and your doctor says you need to start using insulin, it does not mean you're failing to take care of your diabetes properly. It only means that your body is gradually starting to produce less insulin, and oral medications are not any longer adequate to keep your blood glucose at normal levels. Using insulin to get your blood glucose to a healthy level is a good thing, not a bad one.
- If you are starting new medications, ask your doctor the following questions: How many pills should I take? At what times should I take them? Should I take my medicine on an empty stomach or after eating? What if I forget to take my medicine and remember later? What side effects could I have? What should I do if I suffer side effects? Will my diabetes medication cause problems with any of my other medications?
- Call your doctor if you think you are having side effects from your medicine. Do not stop taking it unless the doctor tells you to. Remember, your medicine will work best if you also make healthy changes to how you eat and if you are daily active.
- Medications cannot replace a healthy diet and regular exercise. That’s why diabetes treatment depends more on you than on the medications prescribed by your doctor. The medications will not be of benefit if they are not combined with healthy eating, as they will lose a great part of their effectiveness.
- The strength and efficacy of oral medications does not depend on the cost of the drug.
- Unused insulin should be kept in the fridge and must not be frozen. Used insulin should be kept at room temperature.

Tests and blood sugar measurement:

- Blood glucose levels are usually tested before and after meals, at bedtime, and prior to exercise.
- Home blood sugar testing is an important part of controlling your diabetes.
- Checking your blood sugar tells you how food, activity and medicine are affecting your blood glucose and helps you make sure your blood glucose isn’t going too high or too low.
- Keep a record of your measurements. Talk to your doctor about how often you should check your blood glucose and about what your target numbers should be. He will advise you based on your type of diabetes and treatment plan. Those on insulin should test at least 3 times a day while those who are not should decide on the frequency with their doctor.
- As part of your day-to-day routine, self-monitoring of blood glucose can help with necessary lifestyle and treatment choices and monitor for symptoms of hypo- or hyperglycemia. Monitoring can also help your doctor to alter treatment which in order to prevent any long-term complications from developing.
- The way you feel is not always a good or accurate guide to what is really happening. Test regularly and not only when you feel bad!

- Consider purchasing a glucometer at home if you can. It helps you follow up on your blood sugar levels and adapt yourself to treatment based on the results.
- It is recommended to take the HbA1c test every 3 months and take a diabetic eye exam, a urine test, and measure your kidney function yearly even if you have no complaints.
- Treatment should not be stopped prior to blood sugar measurement or before taking any lab tests.
- Blood glucose testing is more informative and safer than urine testing.

Foot care:

- Diabetes causes poor blood flow in the feet and legs. But you can avoid some of the things that cause poor circulation such as smoking, blood pressure and cholesterol.
- Proper shoes should be and seemingly minor skin injuries should be attended to promptly avoid serious infections.
- Ulcers may not hurt. Yet every ulcer should be immediately seen by your doctor in order to avoid infections that can lead to the loss of a limb.
- Diabetics are far more likely to have a foot or leg amputated than other people. But it can be prevented by regular diabetes care.
- The best way to take care of your feet is to look at them and wash them every day.

Diabetes complications:

- Poorly controlled diabetes accelerates hardening of arteries (atherosclerosis), which may lead to coronary heart disease, strokes, and foot problems.
- Poorly controlled diabetes can lead to eye damage, vision impairment, and dental problems.
- Poorly controlled diabetes can cause kidney damage eventually requiring dialysis.
- Poorly controlled diabetes can cause nerve damage leading to loss of sensation in the feet. Patients then fail to notice foot injuries which may lead to serious infections necessitating surgical amputation of the feet or toes.
- Diabetes complications can be prevented or slowed by better control of blood sugar levels.
- Reducing your HbA1c levels results in a significant decrease in the relative risk of foot problems. Further, aggressive control of elevated blood sugar levels decreases the complications of the kidneys, nerves, eyes, and heart. Aggressive control means achieving fasting glucose levels between 70-120 mg/dl; glucose levels of less than 160 mg/dl after meals; and HbA1c levels below 7%.

Reminders:

- Commit to seeing your doctor regularly at the preset times. Also make sure to see your dentist every six months.
- Measure your blood glucose regularly at home. If you do not have a device at home, then go to the nearest pharmacy or come to MUST hospital once a week. Do not forget to record your measurements.
- Make sure to take the right medication or insulin dose at the right preset times. Do not be careless with regard to medication times and avoid skipping doses.
- If you are on insulin, make sure to adjust your insulin dose based on your blood sugar readings if so instructed by your doctor.

- Do not forget to ask your doctor about possible side effects of the medications you take and what should be done in case you experience any of them.
- Observing your body weight is a necessary element in monitoring your blood sugar levels. Do not forget to check your body weight either once a week or at least once a month. You should always know what your current weight is, what your ideal weight is, and the biggest weight you've ever reached.

<u>Warnings:</u>

- If you have diabetes, your chances of having a stroke are higher than those who do not have diabetes.
- Careful while exercising if you have other diseases such as high blood pressure or if you suffer from diabetes complications. Consult with your doctor about the appropriate types and durations of exercise for you.
- Obesity is the biggest enemy to your diabetes! So keep a healthy diet and stay at the normal body weight set by your doctor.
- Lack of diabetes care may lead to injury of the small blood vessels which affects the eyes, kidneys and nerves.
- Careful! Taking your medication or insulin on time does not mean you can eat what you like.
- Careful! Obesity is not a sign of good health and social prosperity.
- Careful! Placing your feet near the fire in cold weather does not improve blood flow.

<u>Advice:</u>

- Always carry something that will quickly raise your blood glucose like juice or glucose tabs, especially if you are walking or exerting physical effort.
- If you sit for long hours at work or at home, try to get up once every hour and take a quick walk around the room.
- Do not stop taking your medications if you feel better or worse. Talk to your doctor about the way you feel and what you should do next.
- Ask your doctor about possible diabetes complications. Learning about your disease is an essential step to actively engage in your treatment process.
- If you're having trouble managing your diabetes, involve your doctor or a family member. They could provide you with great help and support.
- Ask your doctor about what you should do in cases of hyper- or hypoglycemia.
- Exercise regularly as instructed by your doctor. Ask your doctor about proper exercises for you and about the benefits of exercise to your diabetes.
- Unless otherwise instructed by your doctor, a good exercise schedule is 4-5 times a week 15-30 minutes a day.
- Ask your doctor about exceptional cases when you <u>must</u> go see him.
- Have the will to control your diabetes so that you won't suffer its complications.
- If you take an overdose of insulin then start to sweat and feel weak, you should drink some juice.
- Depend on yourself in order to get better. For instance, follow a healthy diet, exercise daily, and of course quit smoking if you are a smoker. Your target should be fewer medications and more exercise and healthy foods.
- Daily movement is necessary for all and not just for diabetics. It helps you lose weight and strengthens the heart vessels.

- It is recommended to self-monitor your blood sugar at home or at the nearest pharmacy. The lab test is then meant to ensure the accuracy of your device or your ability to self-check.
- Always keep a pack of urine testing strips at home!
- Always keep an insulin dose at home for emergencies.

Hyper- and hypoglycemia:

- High blood sugar measurements occur when you do not commit to your medication times, your meal plan or your exercise schedule and include: fasting glucose > 130, 2 hours after meals > 180, random > 180, and HbA1c > 7 %. They are associated with increased thirst and urination.
- Low blood sugar (hypoglycemia) can occur due to excessive use of insulin or medications, skipped meals, or excessive physical exertion. Measurements are then $\leq$ 70 associated with shakiness and increased sweating.
- Severely high or low blood sugar levels can lead to a diabetic coma.
- Glucagon can be lifesaving. Every patient with diabetes who has a history of hypoglycemia (particularly those on insulin) should have a glucagon kit at home.
- It is recommended to drink some juice or eat something sweet when you experience a sudden blood sugar drop. It is also advised to drink a lot of water and take an emergency dose of insulin when your blood sugar levels are suddenly elevated. Ask your doctor about this emergency insulin dose. And do not worry if you are normally not on insulin! Insulin is not addictive so taking an emergency dose does not mean that you will have to start taking insulin. But careful! If your suddenly elevated blood sugar levels are accompanied with excessive vomiting, then you have to go to the hospital immediately!

Care during illness:

- Infection such as cold or flu can raise your blood sugar levels.
- Check your blood glucose more often if you have a cold or flu.
- Medications should not be stopped during illness unless so advised by your doctor.
- Urine ketones should be tested during intercurrent illnesses and periods of poor control.

Diabetes effects on social and personal life:

- If your diabetes causes you fear, anger or stress, remember that this is normal. You can talk to your doctor, family or friends and include them in your healthy lifestyle. You can also invite them to attend your doctor’s appointments with you.
- Have the will to commit to your meal plan even when eating with others that are not diabetic.
- Have the will to control your diabetes so that it won’t stop you from doing the things you like.
- Diabetes is not an obstacle to normal life and marriage.

برنامج التوعية و المتابعة لمرضي السكر DiabAwPro

"ملحق النظام الغذائي المقترح"

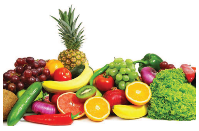

الإفطار:

- فول مدمس (٢ ملعقة) أو بيضة مسلوقة
- ٥٠ – ١٠٠ جم جبن أبيض قليل الدسم أو ٣٠ جم جودة أو فلمنك
- كوب زبادي أو ٢/٣ كوب لبن (١٢٠ مل) نصف أو منزوع الدسم
- ربع رغيف خبز بلدي أو ١ توست رجيم
- طماطم أو خيار أو ثمرة فاكهة

الغداء:

- اللحوم: ١٠٠ جم لحم أحمر أو فراخ أو ٢٠٠ جم سمك منزوعة الجلد و الدهون و يفضل أن تكون مسلوقة أو مشوية
- النشويات: ٣/٤ رغيف خبز بلدي أو ٦ ملاعق أرز أو ٦ ملاعق مكرونة
- الخضروات: كرنب – قرنبيط – باذنجان – فاصوليا خضراء – ملوخية – سبانخ – كوسة – خرشوف – بامية – خبيزة
- فاكهة طازجة

العشاء:

- ١٠٠ جم جبن أبيض قليل الدسم
- فول مدمس (٢ ملعقة) أو طبق عدس
- كوب زبادي نصف أو منزوع الدسم
- نصف رغيف خبز بلدي أو ٢ توست رجيم

الفاكهة (نوع واحد في اليوم):

- تفاح (١ ثمرة) – برتقال (٢ ثمرة) – جوافة (٢ ثمرة) – كمثري (٢ ثمرة) – يوسفي (٢ ثمرة) – خوخ (٢ ثمرة) – بطيخ أو كنتلوب (٢ قطعة متوسطة الحجم) – برقوق أو مشمش (٣ ثمرات)

مأكولات مسموح بها دون تحفظ:

- الفلفل الأخضر – الخس – الفجل – الخيار – الكرفس – البقدونس – الجرجير – البصل
- التوابل ما عدا الملح فيراعي عدم الإكثار منه

الممنوعات (لا تضاف للوجبات و يمكن تناولها حسب توجيهات الطبيب):

- الحلويات الشامية و المكسرات و حلوي المولد
- العسل الأسود – الشوكولاتة – الحلاوة الطحينية – الطحينة
- التورتة – الجاتوه – المربي
- المشروبات الغازية
- العنب – التين – المانجو – البلح

و تراعي مراجعة الطبيب المتابع في حالة الرغبة في اضافة أنواع أخري الي الوجبات المقترحة أو الاصابة بأمراض أخري أو عدم توفر أحد محتويات تلك القائمة لاستبدالها بما يلائم حالة المريض

Diabetes Awareness Program

"The proposed diet leaflet"

Breakfast:

- Fava beans (2 tablespoons) or a boiled egg
- 50-100 g low-fat white cheese or 30 g Gouda
- A cup of yogurt or 2/3 cup of low-fat or skimmed milk (120 ml)
- A quarter of a loaf of pita or a piece of diet toast
- Tomato or cucumber or a piece of fruit

Lunch:

- Meat: 100 g red meat or chicken or 200 g fish without skin and fat, preferably boiled or grilled
- Starches: 3/4 loaf of pita or 6 tablespoons of rice or pasta
- Vegetables: cabbage - cauliflower - eggplants - green beans - Molokhia - spinach - zucchini - artichoke - okra - mallows
- Fresh fruit

Dinner:

- 100 g low-fat white cheese
- Fava beans (2 tablespoons) or a lentil dish
- A cup of low-fat or fat-free yogurt
- Half a loaf of pita or 2 pieces of diet toast

Fruit (one type per day)

- Apple (1 fruit) - Orange (2 fruits) - Guava (2 fruits) - Pear (2 fruits) - Tangerine (2 fruits) - Peaches (2 fruits) - Watermelon or Cantaloupe (2 medium-sized pieces) - plum or apricot (3 fruits)

Foods allowed without reservation:

- Green paprika - lettuce - radish - cucumber - celery - parsley - watercress - onions
- Spices except salt

Foods not allowed (not added to the meals but could be taken as directed by your doctor)

- Sweets, nuts and candy
- Molasses - chocolate - halva - tahini
- Pies - cakes - jam
- Soda drinks
- Grapes - figs - mango - dates

Please refer to your doctor in case you wish to add other food types to the suggested meals, if you suffer from other diseases, or if you could not find any of the listed items in order to suitably replace it.
