# Supplementary material for: SMS education for the promotion of diabetes self-management in low & middle income countries: a pilot randomized controlled trial in Egypt
Source: BMC Public Health. 2017 Dec 19;17:962. doi: 10.1186/s12889-017-4973-5 (PMC5735794; doi:10.1186/s12889-017-4973-5)
Supplement: Supplementary file 3 — SMS content; the educational SMS messages listed by category and followed by their English translation. (PDF 121 kb) [file 12889_2017_4973_MOESM3_ESM.pdf]

برنامج التوعية و المتابعة لمرضي السكر

"الرسائل النصية"

- مرحبا بكم في برنامج التوعية و المتابعة لمرضي السكر! رجاء اتباع الارشادات للحفاظ علي نسبة السكر في الدم

<u>معلومات عامة عن مرض السكر و تأثيره علي حياتك الشخصية و الاجتماعية (ترسل يوم السبت):</u>

1. التعلم عن مرض السكر جزء حيوي من العلاج! اسأل طبيبك عن أي شيء ترغب في معرفته عن السكر
2. حافظ علي السكر الصائم بين ٧٠ و ١٣٠ و العشوائي و بعد الأكل بساعتين تحت ١٨٠ و الهيموجلوبين السكري حول ٧% . نتيجة التحليل متاحة بالعيادة
3. انتبه! السكر مرض خطير لكن التحكم السليم فيه يمنع أو يؤخر ظهور مضاعفات
4. السكر ليس معديا و لكنه مزمن. فلا تتوقع الشفاء بعد فترة من العلاج. بل يجب عليك التعايش معه و التحكم فيه
5. الهيموجلوبين السكري هو مقياس لمستوي السكر في الدم للثلاث أشهر الماضية
6. اسأل طبيبك عن الحالات الاستثنائية التي يجب عليك زيارته ان واجهتها
7. أشرك عائلتك في نمط حياتك الصحي و ادعوهم لحضور مواعيد الطبيب معك فقد يوفر لك ذلك دعما كبيرا!
8. تحدث مع طبيبك أو أفراد العائلة إن سبب لك السكر خوف أو غضب أو توتر
9. مرض السكر نوعان كلاهما ليس بعقبة في طريق الحياة الطبيعية أو الزواج! إسأل طبيبك عن النوع الذي تعاني منه
10. حافظ علي وزن صحي! فالسمنة ليست علامة للصحة الجيدة أو الرخاء الاجتماعي
11. فلتكن لديك العزيمة للسيطرة على السكر حتي لا يمنعك من فعل ما تحب!
12. المتابعة الذاتية إستراتيجية أساسية! فهي ترفع من جودة وسلامة العلاج

<u>الغذاء (ترسل يوم الأحد):</u>

1. الحفاظ على نظام غذائي صحي عنصر أساسي للتحكم في مستويات السكر في الدم
2. ليس هناك غذاء خاص لمرضي السكر! فالغذاء الصحي لمرضي السكر هو نفسه الغذاء الصحي لأي شخص
3. الغذاء الصحي قليل الدهون معتدل في الملح والسكر و يحتوي علي البروتين الطري والخضروات و النشويات بكمية معتدلة و الفاكهة
4. انتبه! يجب الحفاظ علي وزن صحي مثالي فزيادة الوزن هي العدو الاول للسكر!
5. ينصح بالتنويع في الطعام وعدم الإكثار من نوع واحد من الغذاء وعدم تناول الكثير من الطعام في مرة واحدة!
6. نصيحة! باعد بين وجبات الطعام بالتساوي على مدار اليوم ولا تتخطي أي وجبة
7. إن شعرت بالجوع بين الوجبات فتناول وجبة خفيفة. إتفق مع طبيبك علي الوجبات الخفيفة المناسبة لك
8. اسأل طبيبك عن كيفية قياس كمية السكريات في الوجبات التي تتناولها
9. احذر! أخذ الدواء أو الأنسولين بانتظام لا يعني أبدا أن تأكل كما تريد
10. الفاكهة مفيدة للصحة و لكن الاكثار منها قد يضر! إسأل طبيبك عن الأنواع والكميات الصحية المناسبة لك
11. من الأفضل دائما أن تأكل بالبيت! و لكن إن أكلت بالخارج فتذكر أساسيات الغذاء الصحي
12. الحلويات ليست ممنوعة إن كانت في حدود خطة الغذاء الصحية و إلى جانب ممارسة الرياضة!

<u>الحركة و الرياضة (ترسل يوم الاثنين):</u>

1. تذكر! أي نوع من النشاط البدني يساعد على خفض نسبة السكر في الدم
2. من أنواع النشاط المشي والسباحة و استخدام الدرج بدلا من المصعد ... الخ. اسأل طبيبك عن الأنشطة المناسبة لك!
3. حاول أن تمارس النشاط البدني ٣٠ دقيقة يوميا
4. يمكنك تقسيم نشاطك اليومي بالمشي ١٠ دقائق قبل أو بعد وجبات الطعام بدلا من ٣٠ دقيقة دفعة واحدة
5. إن كنت تجلس لساعات طويلة في العمل أو المنزل، فحاول النهوض مرة كل ساعة و المشي حول الغرفة
6. المشي وسيلة رائعة للحصول علي اللياقة! اسأل طبيبك عن جدول المشي الجيد و ادعو الآخرين للمشي معك لجعله أكثر متعة!

7. المشي يحسن ضغط الدم والكولسترول والتوتر والاكتئاب و يساعد علي فقدان الوزن ويقلل من خطر الإصابة بأمراض القلب
8. من المهم شرب الماء و إرتداء الأحذية المناسبة عند المشى أو بذل الجهد!
9. احمل دائما أحد السكريات التي ترفع السكر سريعا عند الخروج أو بذل الجهد
10. من الأفضل ممارسة الرياضة في المناخ المعتدل وليس في شدة الحر أو البرد!
11. احذر! لا تجهد نفسك أثناء ممارسة الرياضة. إسأل طبيبك عن أنواع وفترات التمارين المناسبة لك
12. إسأل طبيبك عن ما يجب فعله حتي لا ينخفض سكرك أثناء المجهود

التدخين، العناية بالقدم، و مضاعفات السكر (ترسل يوم الثلاثاء):

1. لا تدخن! فالتدخين يجعل الشرايين تتصلب بشكل أسرع و يهدد مرض السكر
2. مضاعفات السكر يمكن الوقاية منها أو تأخير الاصابة بها بالتحكم الجيد في مستويات السكر
3. المتابعة المستمرة للسكر و إرتداء الأحذية المناسبة تقيك من البتر الجراحي للقدم أو الساق
4. احذر! التدخين والسكر يضعفان تدفق الدم الي القدمين مما يؤخر شفاء الجروح
5. لا تهمل قرح القدمين حتي ان كانت غير مؤلمة. فيجب زيارة الطبيب ليفحص كل قرحة على الفور!
6. الحفاظ على نسبة طبيعية للهيموجلوبين السكري يقلل خطر الاصابة بمضاعفات
7. احذر! معظم مرضي السكر الذين يحتاجوا الي بتر جراحي للقدم هم من المدخنين
8. نصيحة! إحم قدميك بارتداء الأحذية المناسبة وعدم تجاهل الإصابات الطفيفة!
9. احذر! إهمال السكر قد يؤدي إلى مضاعفات في العين والكلى والقلب والقدمين واللثة والأسنان
10. إعتمد على نفسك من أجل تعديل حالتك الصحية! إتبع خطة الغذاء و مارس الرياضة يوميا و توقف عن التدخين إن كنت تدخن
11. انتبه! أفضل وسيلة لرعاية القدمين هي بالنظر إليهم وغسلهم كل يوم
12. فلتكن لديك العزيمة للسيطرة على السكر حتي لا تصاب بمضاعفاته!

الدواء و اثاره الجانبية (ترسل يوم الأربعاء):

1. لتجنب مضاعفات السكر عليك تناول الدواء أو الانسولين في المواعيد المحددة
2. تذكر! يكون الدواء أكثر فاعلية مع اتباع نظام غذائي صحي وممارسة النشاط البدني
3. يجب أخذ الجرعة الصحيحة للدواء أو الانسولين في الأوقات المحددة و عدم ترك أي جرعة
4. اسأل طبيبك عن ما يجب فعله إن نسيت تناول الدواء وتذكرت لاحقا و ان كان دواء السكر سيسبب أي مشاكل مع أدوية أخري تتناولها
5. تحدث مع طبيبك ان عانيت من الاثار الجانبية للدواء ولا تقرر وقفه بمفردك!
6. حتى لو شعرت بتحسن لا يجب وقف الدواء أو الانسولين ما لم ينصح طبيبك بذلك
7. احذر! لا يجب وقف الدواء عند الاصابة بأمراض أخري ما لم ينصح الطبيب بذلك
8. اعتمادك على نفسك للتحكم بالسكر أهم من اعتمادك علي الدواء أو الإنسولين!
9. إسأل طبيبك عن الأثار الجانبية للدواء ولكن تذكر! الهدف هو: أدوية أقل وزيادة فى التنظيم الغذائي والرياضة
10. انتبه! لا تعتمد قوة وفعالية العلاج علي سعر الدواء
11. إستخدام الانسولين للتحكم في السكر ليس بأمر سيئ و لا يعني أبدا أنك فشلت في التحكم في المرض
12. ينصح لكل مريض سكر الاحتفاظ بجرعة انسولين بالبيت للطوارئ!

التحاليل و قياس السكر (ترسل يوم الخميس):

1. قياس السكر باستمرار بالبيت أو الصيدلية جزء مهم للتحكم في مستويات السكر
2. الأوقات المعتادة لقياس السكر هي قبل و بعد الوجبات و قبل و بعد المجهود و عند النوم
3. قياس السكر بانتظام يخبرك بمدي تأثير الغذاء والرياضة والعلاج على نسبة السكر في الدم
4. سجل نتائج قياس السكر واسأل طبيبك عن النتائج المثالية التي عليك تحقيقها

5. ينصح للمتناولين للأنسولين بقياس السكر ٣ مرات يوميا علي الأقل. أما غير المتناولين فعليهم سؤال الطبيب
6. تذكر! المتابعة الذاتية للسكر تساعدك و طبيبك على مراقبة و تغيير العلاج عند اللزوم لتفادي أي مضاعفات على المدى الطويل
7. ينصح بتحليل الهيموجلوبين السكري كل ٣ شهور وتحليل بول و وظائف كلي سنويا
8. ينصح بعمل فحص لقاع العين كل عام حتي ان كنت لا تشكو من شيء و زيارة طبيب الأسنان كل ٦ شهور
9. انتبه! العلاج لا يجب أن يتوقف قبل قياس السكر أو عمل التحاليل الطبية
10. قم بقياس السكر بانتظام وليس فقط عندما تشعر بسوء! فما تشعر به ليس دائما دليل جيد على ما يحدث
11. قم بقياس السكر أكثر من المعتاد عند الاصابة بالبرد أو الانفلونزا و اختبر الكيتونات في البول
12. جهاز قياس السكر بالبيت يساعدك على متابعة مستويات السكر والتكيف مع العلاج بناء على النتائج

<u>الارتفاع أو الانخفاض فى السكر (ترسل يوم الجمعة):</u>

1. احذر! يرتفع السكر ان زاد تناولك للطعام أو قلت ممارستك للرياضة عن المتفق عليه مع الطبيب أو عند الاصابة بالبرد أوالانفلونزا
2. احذر! ينخفض السكر بسبب الإفراط في استخدام الأنسولين أو الأدوية، أو ترك وجبة من الوجبات المحددة، أو بذل المجهود البدني الزائد
3. احذر! يعتبر السكر مرتفع ان كانت نتائج القياس أعلي من ١٣٠ صائم، ١٨٠ عشوائي أو ساعتين بعد الأكل، و ٧% هيموجلوبين سكري مع عطش و تبول زائد
4. احذر! يعتبر مستوي السكر منخفض ان كان أقل من ٧٠ مع وجود رعشة وعرق بارد
5. احذر! الارتفاع أو الانخفاض الشديد للسكر قد يؤدي الي غيبوبة
6. التحكم الجيد في السكر مهم! فمن الصعب مكافحة الالتهابات مع ارتفاع السكر
7. اسأل طبيبك عن ما يجب فعله في حالات الارتفاع أو الانخفاض الشديد للسكر
8. ينصح بتناول كوب من العصير أو قطعة من الحلوي عند الهبوط المفاجئ للسكر
9. ينصح بشرب الكثير من الماء وأخذ جرعة إستثنائية من الإنسولين عند الإرتفاع المفاجئ للسكر. إسأل طبيبك عن هذه الجرعة!
10. لا تقلق! إن كنت لا تأخذ الانسولين و اضطررت لأخذ جرعة إستثنائية فهذا لا يعني أنك ستأخذه دائما
11. إن كنت تتقيأ كثيرا مع إرتفاع مفاجئ للسكر فإذهب إلي المستشفي علي الفور!
12. الجلوكاجون قد ينقذ حياتك! يجب علي كل مريض سكر، خاصة من يأخذ الانسولين أو ينخفض سكره أحيانا، أن يحتفظ بطقم الجلوكاجون بالمنزل

Diabetes Awareness Program

"SMS content"

- Welcome to MUST diabetes awareness program! Please follow the instructions in order to keep your blood glucose levels normal.

Diabetes knowledge and effects on social and personal life (sent on Saturdays):

1. Learning about diabetes is a vital part of treatment. Ask your doctor if there is anything you would like to know about diabetes.
2. Keep your fasting/pre-meals sugar levels between 70 and 130, random/2 hours post-meals under 180, and your HbA1c levels around 7%.
3. Attention! Diabetes is a serious disease but managing it properly can prevent or delay its complications.
4. Diabetes is not contagious but it is a chronic disease. Cure is not to be expected after a short course of treatment. You have to learn to live with and control it.
5. HbA1c is a measure of your blood glucose levels in the past 3 months.
6. Ask your doctor about exceptional cases when you must go see him.
7. Include your family members in your new healthy lifestyle and invite them to join you for your doctor's appointments. Involving them can provide you with great support!
8. Talk to your doctor or family members if your diabetes causes you fear, stress or anger.
9. There are two types of diabetes; both are not an obstacle to normal life and marriage. Ask your doctor about the type of diabetes you have.
10. Keep a healthy weight! Obesity is not a sign of good health and social prosperity.
11. Have the will to control your diabetes so that it won't stop you from doing the things you like.
12. Self-monitoring is an essential strategy! It improves the quality and safety of therapy.

Diet (sent on Sundays):

1. Keeping a healthy diet is essential for controlling your blood sugar levels.
2. There is no special diabetic food! A healthy meal plan for diabetics is generally the same as a healthy diet for anyone.
3. A healthy meal is low in fat, moderate in salt and sugar, and based on lean protein, vegetables, starches in moderate quantities, and fruit.
4. Careful! It is necessary to stay at a healthy body weight. Obesity is the greatest enemy to your diabetes!
5. Tip! Try to eat a variety of food, avoid eating too much of one type, and do not eat too much at once.
6. Tip! Try to space your meals evenly throughout the day and try not to skip any meal.
7. Take a snack if you feel hungry between meals. Ask your doctor about possible snacks for you.
8. Ask your doctor about how to measure the quantity of sugar in your meals.
9. Careful! Taking your medication or insulin regularly does not mean you can eat what you like.

10. Fruits are healthy, but you cannot have as much as you wish! Ask your doctor about the right portions and types of fruit for you.
11. It is always healthier to eat at home than outside. But if you do, remember to follow the basics of a healthy diet.
12. Sweets are not off limits if part of a healthy meal plan and combined with exercise!

Physical activity (sent on Mondays):

1. Remember! Any type of physical activity you do helps lower your blood glucose!
2. Examples of different types of physical activity include walking, swimming, taking the stairs instead of an elevator, etc. Talk to your doctor if you have questions about which activities are right for you.
3. Try to be active 30 mins per day.
4. You can split up your activity, e.g. walk 10 mins before or after meals instead of 30 mins at once.
5. If you sit for long hours at work or at home, try to get up once every hour and take a quick walk around the room.
6. Walking is a great way to get fit! Ask your doctor about a good walking schedule for you and invite others to join! It makes it more pleasant!
7. Walking improves blood pressure, cholesterol, stress, and depression, promotes weight loss and reduces your risk of heart disease.
8. It is important to stay hydrated and wear proper shoes while walking or exercising!
9. Always carry a strong acting sugar that will quickly raise your blood glucose especially when walking or leaving the house.
10. It is better to exercise in moderate climate and not in extreme heat or cold.
11. Careful! Do not exhaust yourself during exercise. Ask your doctor about appropriate types and durations of exercise for you.
12. Ask your doctor about what you should do so that your blood sugar won’t drop during physical exertion.

Smoking, foot care and diabetes complications (sent on Tuesdays):

1. Don't smoke! Smoking makes arteries harden faster and threatens your diabetes.
2. Diabetes complications can be prevented or slowed by good control of sugar levels.
3. Regular diabetes care and proper footwear protect you from having a foot or leg amputated.
4. Careful! Smoking as well as diabetes cause decreased blood flow to the feet which makes wounds heal slowly.
5. Careful! Do not neglect foot ulcers even if they do not hurt. Every foot ulcer should be seen by your doctor!
6. Keeping your HbA1c at the normal levels reduces your risk of suffering diabetes complications.
7. Careful! Many diabetics who need amputations are smokers.
8. Tip! Protect your feet by wearing proper shoes and do not ignore minor injuries!
9. Careful! Uncontrolled diabetes can lead to complications in the eyes, kidneys, heart, feet, gums and teeth.

10. Depend on yourself in order to get better! Follow a healthy diet, exercise daily, and quit smoking if you are a smoker.
11. The best way to take care of your feet is to look at them and wash them every day.
12. Have the will to control your diabetes so that you won't suffer its complications.

Medications and side effects (sent on Wednesdays):

1. Taking your medications on time is essential to avoid diabetes complications.
2. Remember, your medicine will work best if you also make healthy changes to how you eat and if you are daily active.
3. Make sure to take the right medication/insulin dose at the right preset times and avoid skipping doses.
4. Ask your doctor about what you should do if you forget to take your medicine then remember later and if your diabetes medication will cause problems with any other medications you take.
5. Talk to your doctor if you experience side effects! Do not decide to stop on your own!
6. Even if you feel better, do not stop taking your medication unless your doctor says so.
7. Medications should not be stopped during illness unless so advised by your doctor.
8. Depending on yourself to control your diabetes is more important than relying on medications or insulin!
9. Ask your doctor about the side effects of your medications but remember! Your target should be: Lesser medications and more exercise and healthy eating!
10. Attention! The strength and efficacy of oral medications does not depend on the cost of the drug.
11. Using insulin to get blood glucose levels to a healthy level is not a bad thing and does not necessarily mean you are failing to manage your diabetes!
12. Always keep an insulin dose at home for emergencies.

Tests and blood sugar measurement (sent on Thursdays):

1. Frequent blood sugar testing at home or pharmacy is an important part of controlling blood sugar.
2. Usual blood sugar testing times are before and after meals, before and after exercise and at bed time.
3. Checking your blood glucose regularly is one way you can know how food, activity and medicine affect your blood glucose.
4. Keep a record of your blood glucose measurements and ask your doctor about your target numbers.
5. ADA guidelines indicate that those on insulin should test at least 3 times a day while those who are not should decide on the frequency with their doctor.
6. Self-monitoring can help you and your doctor to alter treatment which in turn can help prevent any long-term complications from developing.
7. It is recommended for diabetics to take the HbA1c test every 3 months, and take a urine test and measure their kidney function yearly.
8. It is recommended for diabetics to take a diabetic eye exam every year even if they have no complaints and see their dentist every six months.

9. Treatment should not be stopped prior to blood sugar measurement or before taking any lab tests.
10. Test your blood sugar regularly and not just when you feel bad! The way you feel is not always a good or accurate guide to what is happening.
11. Check your blood glucose more often if you have a cold or flu and test for urine ketones
12. Having a glucometer at home helps you follow up on your blood sugar levels and adapt yourself to treatment based on the results.

Hyper- and hypoglycemia (sent on Fridays):

1. Careful! Eating more or exercising less than planned, or having a cold or flu leads to increased blood sugar levels.
2. Careful! Low blood sugar (hypoglycemia) can occur due to excessive use of insulin or medications, skipped meals, or excessive physical exertion.
3. Careful! High blood sugar measurements include: fasting glucose $\geq$ 130, 2 hours after meals $\geq$ 180, random $\geq$ 180, and HbA1c $\geq$ 7 % with increased thirst and urination.
4. Careful! Hypoglycemia is characterized by blood sugar levels below 70, sweating and shakiness.
5. Careful! Severely high or low blood sugar levels can lead to diabetic coma.
6. Good diabetes control is important! High blood glucose levels makes it hard to fight infection.
7. Ask your doctor about what you should do in case of hyper or hypoglycemia.
8. It is recommended to drink some juice or eat something sweet when you experience a sudden blood sugar drop.
9. It is advised to drink a lot of water and take an emergency dose of insulin when your blood sugar levels are suddenly elevated. Ask your doctor about this emergency insulin dose.
10. Do not worry if you are not normally on insulin! Taking an emergency dose does not mean that you will have to start taking insulin.
11. If you experience a sudden increase in your blood sugar levels accompanied with excessive vomiting, then go to the hospital immediately!
12. Glucagon can be lifesaving! Every patient with diabetes who has a history of hypoglycemia (particularly those on insulin) should have a glucagon kit at home.
